# Mitochondrial Dynamics and Mitophagy in Cardiometabolic Disease

**DOI:** 10.3389/fcvm.2022.917135

**Published:** 2022-06-17

**Authors:** Jianguo Lin, Jinlong Duan, Qingqing Wang, Siyu Xu, Simin Zhou, Kuiwu Yao

**Affiliations:** ^1^Guang'anmen Hospital, China Academy of Chinese Medical Sciences, Beijing, China; ^2^Tianjin University of Traditional Chinese Medicine, Tianjin, China; ^3^Beijing University of Chinese Medicine, Beijing, China; ^4^Eye Hospital China Academy of Chinese Medical Sciences, Beijing, China

**Keywords:** mitochondrial dynamics, cardiometabolic disease, mitophagy, mitochondrial fission, mitochondrial fusion, diabetic cardiomyopathy, myocardial infarction

## Abstract

Mitochondria play a key role in cellular metabolism. Mitochondrial dynamics (fusion and fission) and mitophagy, are critical to mitochondrial function. Fusion allows organelles to share metabolites, proteins, and mitochondrial DNA, promoting complementarity between damaged mitochondria. Fission increases the number of mitochondria to ensure that they are passed on to their offspring during mitosis. Mitophagy is a process of selective removal of excess or damaged mitochondria that helps improve energy metabolism. Cardiometabolic disease is characterized by mitochondrial dysfunction, high production of reactive oxygen species, increased inflammatory response, and low levels of ATP. Cardiometabolic disease is closely related to mitochondrial dynamics and mitophagy. This paper reviewed the mechanisms of mitochondrial dynamics and mitophagy (focus on MFN1, MFN2, OPA1, DRP1, and PINK1 proteins) and their roles in diabetic cardiomyopathy, myocardial infarction, cardiac hypertrophy, heart failure, atherosclerosis, and obesity.

## Introduction

Cardiometabolic disease (CMD) is a clinical syndrome caused by genes (heredity, environment, behavior) and metabolic drivers (abnormal obesity, abnormal blood glucose, metabolic syndrome characteristics), including hypertension, diabetes, dyslipidemia, coronary heart disease, stroke, and other diseases (1, 2). The incidence of CMD increases gradually with the increase of age, which is the primary cause of death and disease burden of the global population. Unreasonable diet, lack of exercise, smoking, and excessive drinking are the main risk factors for the sharp increase in CMD (3). At present, pathogenesis, risk assessment, and treatment of CMD remain unclear. One of the most promising treatments for CMD is to improve cardiac metabolism and energy supply (4, 5).

The heart has a very high energy requirement, which must continuously produce large amounts of ATP through the metabolism of various fuels (fatty acids, glucose, lactic acid, pyruvate, and amino acids) to maintain systolic function. Mitochondrial oxidative phosphorylation (OXPHOS) contributes 95% of cardiac ATP requirements and glycolysis provides the remaining 5% (6). Cardiac energy disturbance is an important cause of most CMD (7). The ability of the heart to switch between different energy substrates is known as metabolic flexibility (8). As insulin resistance develops, the metabolic flexibility of the heart gradually decreases, making myocardial energy production largely dependent on fatty acid oxidation. This shift leads to increased uptake and accumulation of lipids in the heart, resulting in lipotoxicity. At the same time, glycolytic intermediates accumulate in the heart due to the unavailability of glucose, producing glucotoxicity (9). By improving mitochondrial homeostasis and converting metabolic pathways, it is beneficial to support the homeostasis of the body environment and promote the change of cell phenotype, thereby improving the pathological mechanism of CMD (10, 11).

The endosymbiosis theory suggests that mitochondria are bacteria that are engulfed by primitive eukaryotes. This bacterium was symbiotic with eukaryotes and became mitochondria through evolution during the long symbiosis (12). Mitochondria are the two membrane-encapsulated organelles present in most cells and are the main sites for the aerobic respiration of cells. Mitochondria make up about one-third of the volume of adult cardiomyocytes and are the primary energy source for the coupling of excitatory contractions in the heart (13). Mitochondria play an important role in sustaining cardiology and physiology as metabolic and signal transduction centers, involved in many important biological processes such as OXPHOS production of ATP, fatty acid oxidation, calcium homeostasis, phospholipid synthesis, production, and maintenance of reactive oxygen species (ROS), and iron-sulfur cluster biosynthesis (14). In recent years, more and more evidence showed that mitochondria and CMD are closely related. Mitochondrial dysfunction can induce ROS production, activate DNA damage responses, lead to cardiac cell cycle arrest, and ultimately lead to fatal cardiomyopathy (15). Mitochondrial Ca^2+^ is the basic substance that activates the mitochondrial respiratory chain complex and ATP production and regulates the key mitochondrial dehydrogenase activity. When mitochondrial calcium homeostasis is unbalanced, mitochondrial bioenergetics are damaged, leading to the occurrence of diabetic heart disease (16).

Given the unique and highly dynamic structure of mitochondria in the heart and intimate links between mitochondria and CMD homeostasis in physiology and pathology. In this review, we focus on the mitochondrial fusion, fission, and mitophagy in CMD (with a focus on diabetic cardiomyopathy, myocardial infarction, heart failure, atherosclerosis, and obesity), and the potential drugs that target mitochondria to treat CMD.

## Introduction to Mitochondria

### Mitochondrial Structure

**Figure 1:**
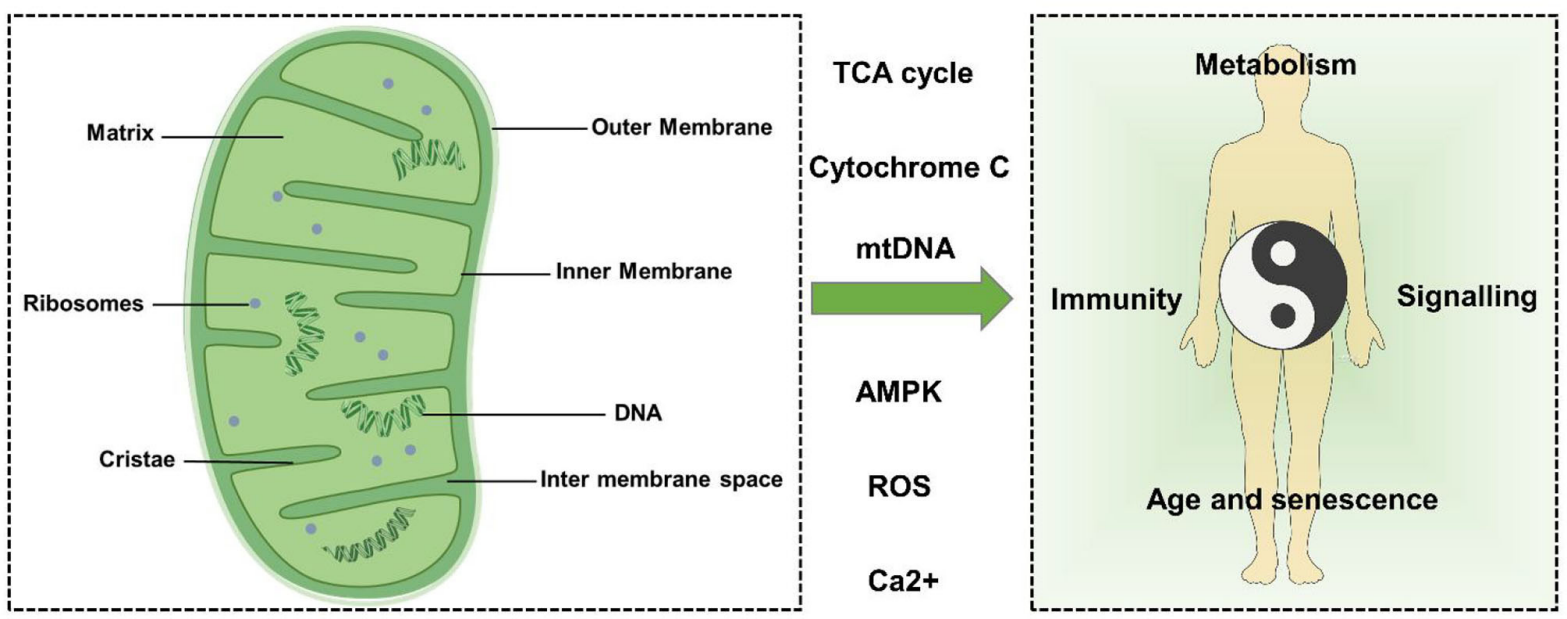
Mitochondrial structure and function. Mitochondria can be divided into four functional regions from outside to inside: outer mitochondrial membrane, intermembrane space, inner mitochondrial membrane, and mitochondrial matrix. Mitochondria can regulate metabolism, signal transduction, immune regulation, cell senescence, and death through TCA cycle metabolites, cytochrome c (Cytc), mtDNA, Ca^2+^, ROS, AMPK, and other factors, thus affecting the balance of the human body.

Mitochondria can be divided into four functional regions from outside to inside: outer mitochondrial membrane (OMM), intermembrane space (IMS), inner mitochondrial membrane (IMM), and mitochondrial matrix (Figure 1).

The OMM is the outermost unit membrane of mitochondria with a thickness of about 6–7 nm. The OMM acts as a diffusion barrier and mediates mitochondrial signaling. The OMM is smooth and usually permeable, restricting diffusion only to molecules greater than ~5,000 Da. Molecules with a molecular weight greater than the above limit require a specific signal sequence for recognition and active transport of mitochondria by translocase of the outer membrane (TOM) (17, 18). Importantly, the OMM is crucial to mitochondrial dynamics because it carries all the molecules involved in mitochondrial fusion and fission.

The IMM is a unit membrane located inside the OMM and surrounding the mitochondrial matrix. The mass ratio of protein to phospholipid in the IMM is about 0.7:0.3 and contains a large amount of cardiolipin. The IMM includes two main sub compartments: the inner boundary membrane (IBM) and mitochondrial cristae. The IBM is the part of the IMM that does not protrude into the matrix but runs parallel to the OMM. The cristae and IBM are connected by narrow tubular or slit structures called cristae junctions (CJS) (19).

Cristae are characteristic folds of the inner membrane that penetrate the matrix. The IBM and cristae are functionally differentiated and have a distinct protein composition. Respiratory chain complexes and proteins involved in iron-sulfur cluster assembly are enriched in cristae membranes, while protein translocation and membrane fusion mechanisms are mainly present in IBM (20–23).

The mitochondrial lumen (named “matrix”) is the inner space enclosed by IMM, which contains many proteins such as enzymes involved in biochemical reactions such as tricarboxylic acid cycle, fatty acid oxidation, and amino acid degradation (24). In addition, the matrix also contains mitochondrial DNA (mtDNA), RNA, and mitochondrial ribosomes (25).

Human mtDNA is a double-stranded circular molecule of 16,569 base pairs with a molecular mass of 107 Da (26). Maintenance of mtDNA stability and integrity is critical for cellular energetics. mtDNA is particularly susceptible to damage, whether by respiration or exogenous contact, which can lead to damage to the base of the DNA. mtDNA is closely related to mitochondrial dynamics (25). When cells undergo apoptosis, mtDNA can be released into the cytoplasm, causing an inflammatory response (27).

### Mitochondrial Fusion and Fission

**Figure 2:**
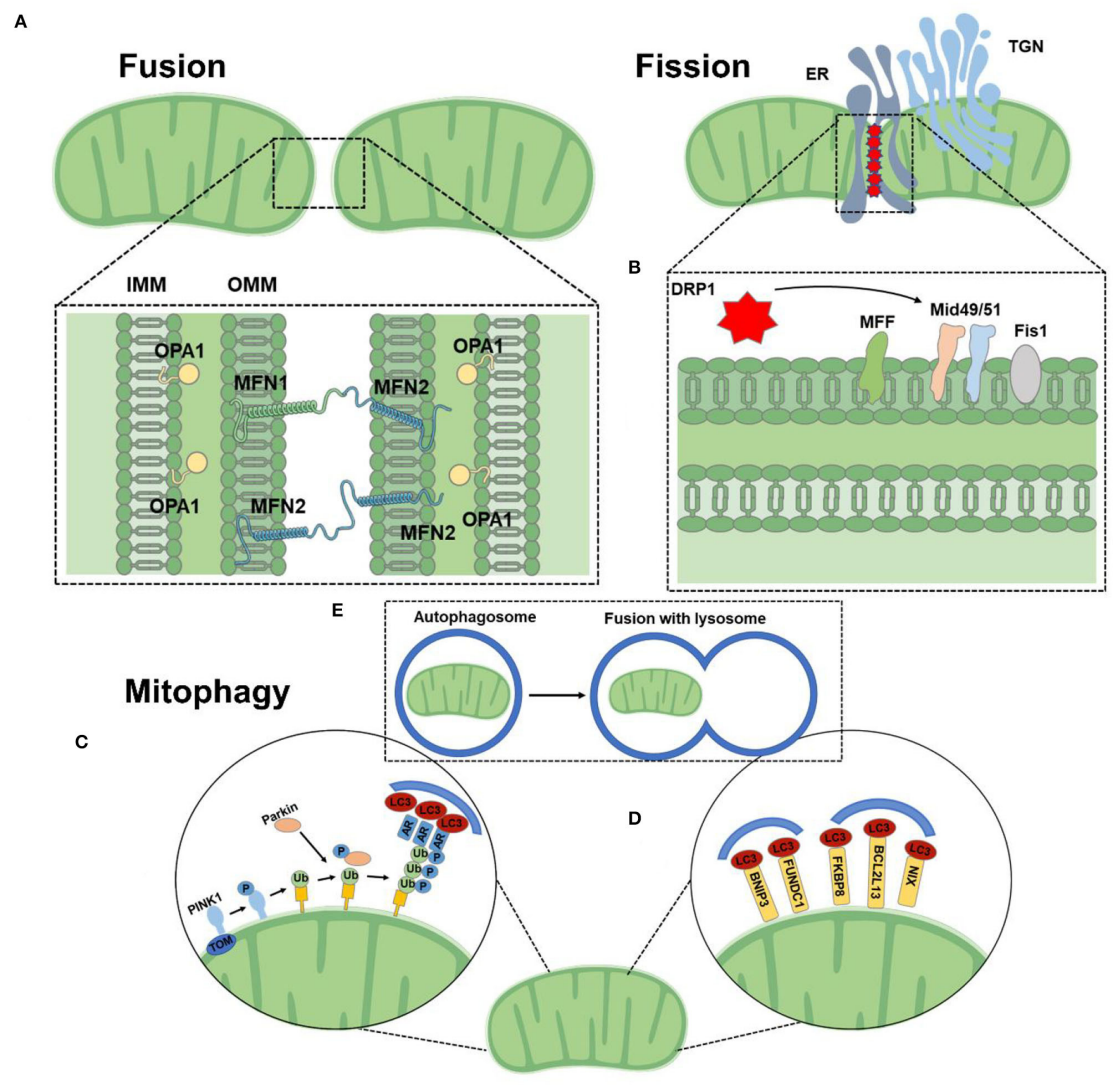
Mitochondrial function. **(A)** Mitochondrial fusion is mediated by homo-and heterotypic interactions between MFN1 and MFN2 at the OMM and OPA1 at the IMM. **(B)** DRP1 binds to its receptors at the OMM at sites of contact with the ER. **(C)** The PINK1/Parkin-dependent pathway: under stress conditions, PINK1 was stable on OMM, which promoted the recruitment of Parkin. Parkin ubiquitizes several outer membrane components. The polyubiquitin chain is then phosphorylated by PINK1 as a “eat me” signal for the autophagy mechanism. Autophagy receptors (AR) recognize phosphorylated polyubiquitin chains on mitochondrial proteins and initiate autophagosome formation by binding to LC3. **(D)** The PINK1/Parkin-independent pathway: mitophagy receptors, such as BNIP3, NIX, FKBP8, and FUNDC1 are located to directly interact with OMM and LC3 to mediate mitochondrial clearance. **(E)** Mitochondria are engulfed by autophagosomes, which fuse with lysosomes and catabolize them.

Homeostasis of mitochondrial dynamics is critical to maintaining cardiac structure and function. Mitochondria are highly dynamic organelles that maintain their shape, distribution, and size through fusion, fission, and mitophagy when cells undergo metabolic or environmental stress (Figure 2). The coordinated cycle of mitochondrial fission and fusion is known as mitochondrial dynamics (28, 29). Mitochondrial fusion allows organelles to share metabolites, proteins, and mtDNA, promoting complementarity between damaged mitochondria (30, 31). Fission increases the number of mitochondria to ensure that they are passed on to their offspring during mitosis. In addition, fission can signal to cells that mitochondria are damaged and need to be removed through mitosis to maintain a healthy mitochondrial network (32–34). The balance of fusion and fission affects the cardiac phenotype. An imbalance between fusion and fission is more detrimental than stopping both processes at the same time. Recently, a study showed that compared to MFN1/MFN2 cardiac knockout or DRP1 cardiac knockout mice, MFN1/MFN2/DRP1 cardiac triple knockout mice survived longer and manifested a unique pathological form of cardiac hypertrophy (35).

The core mechanism of mitochondrial dynamics is regulated by a group of GTPases related to the dynamin family. These proteins can oligomerize and change conformation, driving the remodeling, contraction, shearing, and fusion of mitochondrial membranes (36). Mitochondria are double-membrane organelles and complete fusion requires the merging of the outer and inner membranes. Mitochondrial fusion is a two-step mechanism. The OMM located GTPases mitofusin (MFN) 1 and 2 ensure the OMM fusion, and the IMM located optic atrophy protein 1 (OPA1) is responsible for IMM fusion (37, 38). OMM fusion is mediated by the MFN. MFN form both homo-oligomeric (MFN1-MFN1 or MFN2-MFN2) and hetero-oligomeric (MFN1-MFN2) complexes in trans between apposing mitochondria (39). Mitochondrial fusion is crucial for embryonic development, mice deficient in either MFN1 or MFN2 die in midgestation (40). OPA1 is a large GTPase tethered to the IMM facing the intermembrane space (39). OPA1 is dependent on MFN1 for fusion function, but not MFN2 (37).

Fission is mainly dominated by dynamin-related protein 1 (DRP1) which translocates from the cytoplasm to mitochondria and binds to its OMM partners [mitochondrial fission factor (MFF), mitochondrial dynamics protein of 49 kDa (MID49), MID51, and mitochondrial fission 1 protein (FIS1)] at sites of contact with the endoplasmic reticulum (ER) and actin (14, 41, 42). Following this binding, DRP1 oligomerizes and drives scission. Recently, DRP2 has been found to collaborate to drive mitochondrial fission (43). Loss of DRP1 loss results in highly elongated mitochondria and peroxisome (44). Kageyamae et al. found that DRP1 and Parkin synergistically control the biogenesis and degradation of mitochondria. In the absence of mitochondrial fission mediated by DRP1, mitochondria became defective in mitophagy. This mitophagy defect led to the accumulation of the mitophagy adaptor protein p62 and ubiquitinated proteins on mitochondria in a Parkin-independent manner, increases in cardiac defects, and loss of mitochondrial respiratory competence (45).

### Mitochondrial Biogenesis

Mitochondrial biogenesis is as important as other mitochondrial dynamics (46, 47), but it's almost neglected. Mitochondria especially injured ones actively regulate cell death, which is critical for maintaining cardiac homeostasis (48, 49). Mitochondrial biogenesis is the process by which cells increase mitochondrial mass. Mitochondrial biogenesis requires extensive coordination of both mitochondrial and nuclear genomes. Mitochondrial biogenesis is influenced by a variety of exogenous and endogenous factors such as exercise, caloric restriction, low temperature, oxidative stress, cell division, renewal, and differentiation (50). Mitochondrial biogenesis increases the copy number of mtDNA, the protein subunits of metabolic enzymes, and ultimately leads to greater metabolic capacity. In mammals, mitochondrial biogenesis is primarily regulated by the transcriptional coactivator PGC-1α (46). A large body of evidence suggested that CMD is associated with mitochondrial biogenesis. By regulating the PGC-1α signaling pathway, it is possible to treat diabetic cardiomyopathy, heart failure, obesity, and other diseases (51, 52). There is evidence that the genes involved in mitochondrial fusion (MFN1, OPA1) and fission (DRP1, Fis1) were altered expression in the hearts of PGC-1α/β-deficient mice. Significant mitochondrial structural dysregulation, including breakage and elongation, was observed in the hearts of PGC-1α/β-/– mice, associated with the development of fatal cardiomyopathy (53). In addition, PGC-1α/β-/– mice died shortly after birth with small hearts, bradycardia, intermittent heart block, and a markedly reduced cardiac output (54).

### Mitophagy

Autophagy is an evolutionarily conserved mechanism that segregates superfluous, aging, or damaged cytoplasmic material and deliver it to lysosomes for degradation (55). The main physiological role of autophagy may be to maintain cellular homeostasis in the context of reduced nutrient supply and other metabolic disturbances (56). Autophagy occurs in almost all types of cardiovascular cells, including myocytes, vascular smooth muscle cells (VSMCs), fibroblasts, macrophages, and endothelial cells (57). It has been shown that a measured level of constructive autophagy is beneficial in restoring cardiac homeostasis in the CMD settings; whereas both autophagy impairment and excessive activation can lead to structural and functional dysfunction of the heart (58).

Mitophagy is the selective degradation of mitochondria by autophagy. Mitophagy can promote the turnover of mitochondria, maintain mitochondrial quality, and prevent the accumulation of dysfunctional mitochondria (59). Mitophagy is generally divided into two major functional groups based on the requirements for the kinase PINK1 and the Ub E3 ligase Parkin, often referred to as PINK1/Parkin-dependent (initiating by a loss of mitochondrial membrane potential) and PINK1/Parkin-independent mitophagy (not require loss of the mitochondrial membrane potential) (14). Mitochondrial autophagy is involved in metabolic activity, cell differentiation, apoptosis, and other physiological processes associated with major phenotypic changes, which is an important target for the treatment of CMD (60). Cardiac stress-induced mitophagy helps to remove damaged and dysfunctional mitochondria, thus preventing oxidative damage that could in turn initiate apoptosis and ultimately lead to heart failure (61). Autophagy related 7 (Atg7)-and Parkin-dependent mitophagy plays an essential role in the maintenance of mitochondrial function and protects the heart during the early development of diabetic cardiomyopathy (62). In addition, Atg5 deficiency-mediated mitophagy increases ROS production and NF-κB activity in macrophages, thereby aggravating cardiac inflammation and injury (63). Thus, improving mitophagy may be a novel therapeutic strategy to ameliorate CMD.

## Mitochondrial Dynamics and Mitophagy in Cardiometabolic Diseases

### Diabetic Cardiomyopathy

Diabetic cardiomyopathy (DC) refers to a cardiac disease that occurs in diabetic patients and cannot be explained by hypertensive heart disease, coronary atherosclerotic heart disease, or other cardiac lesions. Diabetic hearts utilize fatty acids as their main source of energy, producing high levels of oxidative stress that can lead to mitochondrial dysfunction (64). More and more evidence suggested that cardiovascular complications of diabetes are concentrated in the mitochondria, which are central to cardiomyocyte damage (65, 66).

**Figure 3:**
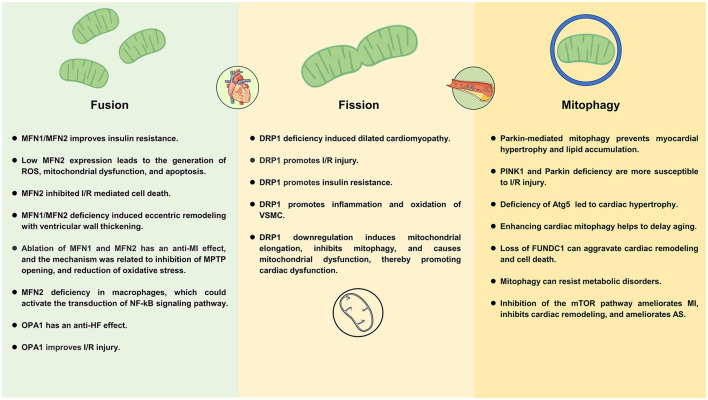
Mechanisms of mitochondrial dynamics and mitophagy in cardiometabolic diseases.

Exposure to excess nutrients promotes the growth of the mitochondrial fission and reduces mitochondrial fusion, which is associated with uncoupled respiration (67). In agreement with this view, in hyperglycemic conditions, mitochondria can induce rapid division through DRP1 signaling, resulting in excessive production of ROS (68). Low MFN2 expression leads to the generation of ROS, mitochondrial dysfunction, and mitochondria-dependent apoptosis, which leading DC (69) (Figure 3). Along this line, a study showed that ablation of MFN2 leads to the development of impaired glucose tolerance, hyperinsulinemia, and insulin resistance (70). Montaigne et al. found that the deterioration of endogenous myocardial contraction during the transition from obesity to diabetes may be related to the deterioration of myocardial mitochondrial function. Furthermore, they indicated diabetes mellitus was associated with cardiac mitochondrial network fragmentation and myocardial MFN1 content was inversely proportional to hemoglobin A1C (71).

A growing number of studies have demonstrated that balancing mitochondrial biogenesis and mitophagy is essential for maintaining cellular metabolism in the DC (72, 73). Mitophagy dysregulation exacerbate high-fat diet (HFD)–induced DC. Tong et al. (62) showed that Parkin-mediated mitophagy protects the heart against HFD-induced cardiac hypertrophy, and diastolic dysfunction and lipid accumulation, furthermore, Tat-Beclin 1 (inducer of autophagy) therapy alleviates cardiac dysfunction induced by HFD. Similarly, Wu et al. (74) found that deficiency of mitophagy receptor FUNDC1 impairs mitochondrial quality and aggravates dietary-induced obesity and metabolic syndrome.

Inflammation plays a key role in the pathogenesis of diabetes mellitus, and inflammatory injury is usually associated with pancreatic β cell dysfunction (75). Mitochondria are negatively affected by pancreatic β cell inflammatory signals, which can lead to impaired mitochondrial dynamics (76, 77). Therefore, strategies that block inflammation and/or protect mitochondrial function are potential treatments for diabetes. Tanajak et al. (78) showed that impaired β cell mitophagy exacerbates hyperglycemia and mitochondrial fragmentation *in vivo* following inflammatory stimuli, and overexpression of CLEC16A (mitophagy regulator whose expression in islets is protective against T1D) can mediate mitophagy and protect β cells from cytokine-mediated death.

Diabetic cardiomyopathy is closely related to diet, exercise, and metabolic changes. Caloric restriction and exercise may be a strategy to treat DC. A study found that caloric restriction is effective in improving metabolic regulation, and attenuating cardiac mitochondrial dysfunction in obese-insulin-resistant rats (79). Furthermore, clinical trials found that exercise improved fat oxidation and insulin sensitivity in obese insulin-resistant adults, and the mechanism was related to the decrease of DRP1 and the increase of MFN1 and MFN2 (80).

Based on the above evidence, we can suggest that inhibition of mitochondrial fission, promotion of mitochondrial fusion, and mitophagy are potential strategies for the treatment of DC. It is worth noting that effective exercise and diet are also a measure to modulate mitochondria.

### Myocardial Infarction

Myocardial infarction (MI) is an irreversible disease of the myocardium, which is based on ischemic hypoxic necrosis of cardiac myocytes (81). Ischemia is generally accompanied by overproduction of ROS, mitochondrial dysfunction, the translocation of Cytc from the mitochondria to the cytoplasm, the opening of the mitochondrial permeability transition pore (mPTP) and inflammatory cascade (82, 83). In this setting, effective mitochondrial homeostasis is important.

Studies demonstrated that ischemia induces mitochondrial fission, inhibition of DRP1 protected cardiomyocytes against ischemia/reperfusion (I/R), inhibited mPTP opening, and reduced the size of MI (84–86). Although it is widely believed that the fused mitochondria function better, however, Ikeda et al. (87) suggested DRP1 plays an essential role in mediating mitophagy in cardiomyocytes, they found that DRP1 downregulation induces mitochondrial elongation, inhibits mitophagy, and causes mitochondrial dysfunction, thereby promoting cardiac dysfunction and increased susceptibility to I/R.

Mdivi-1 pharmacologically inhibits mitochondrial fission by blocking the binding of DRP1 to its receptor. Mdivi-1 given before ischemia exerts cardioprotective effects by reducing the incidence of arrhythmias, decreasing infarct size, improving cardiac mitochondrial function and fragmentation, and reducing cardiac apoptosis (88). Interestingly, another study found negative results, they found that treatment with Mdivi-1 at the start of reperfusion did not reduce MI size or preserve left ventricular function in pig MI model (89). Mitochondrial fission is dependent on ER-mitochondria contact. BAP31, an ER protein, regulates intracellular calcium homeostasis and ER stress (90). Cheng et al. (91) found that silencing Fis1/ BAP31 reduced mitochondrial fission and inhibited JNK activation, which led to a reduction in ROS and promoted cardiomyocyte survival.

Mitochondrial fusion protects stressed cells through two separate mechanisms. First, fusion counteracts the effects of excess mitochondrial fission, thereby limiting fission-induced mitochondrial apoptosis. Second, fusion enhances the timely detection of damaged parts of mitochondria and balances mitochondrial proteins, lipids, metabolites, and mitochondrial DNA (92, 93). Mitochondrial fusion has a protective effect in physiological conditions, but the role of mitochondrial fusion-related proteins in I/R injury remains a hot topic of debate. A study found that MFN2 overexpression significantly inhibited I/R mediated cell death by promoting mitochondrial fusion, regulating glucose metabolism, and oxidative stress (94). Interestingly, another study reported the opposite observation, they showed that ablation of MFN1 and MFN2 genes had a protective effect on myocardial infarction, and the mechanism was related to inhibition of MPTP opening, reduction of oxidative stress, and attenuating mitochondrial Ca^2+^ overload (95). Analogously, MFN1 KO heart mitochondria displayed a normal respiratory repertoire and were not affected by mitochondrial depolarization and their viability was enhanced when attacked by ROS in the form of hydrogen peroxide (96).

Optic atrophy protein 1-related mitochondrial fusion and mitophagy are vital to sustaining mitochondrial homeostasis under stress conditions (97, 98). Le et al. (99) showed that increase in cardiac I/R injury, impairment of dynamic mitochondrial Ca^2+^ uptake, and increased incidence of arrhythmias in Opa1+/- mouse models. Similarly, Zhang et al. (100) demonstrated that manipulation of the AMPK-OPA1-mitochondrial fusion/mitophagy axis *via* melatonin can block cardiomyocyte caspase-9-involved mitochondrial apoptosis, which attenuates I/R injury.

Mitochondrial damage and ROS produced by mitochondrial oxidative stress can act as substrates to induce mitophagy (101). During ischemia, upregulation of mitophagy is considered beneficial (102, 103). For instance, a study showed that Parkin ablation caused a decrease in a severe decrease in mitochondrial respiration, mitochondrial uncoupling, and increased susceptibility to the opening of the permeability transition pore (104). Further, Parkin (–/–) mice were much more sensitive to MI, and Parkin (–/–) myocytes had reduced mitophagy and accumulated swollen, dysfunctional mitochondria after the infarction (105). PINK1 protein is highly expressed in the myocardium. PINK1–/– mice had larger MI and were more susceptible to I/R injury, which is associated with impaired mitochondrial function (106). Therefore, induction of autophagy through activation of the Pink1/Parkin pathway could exert a protective effect against MI (107).

The mTOR pathway is a well-known upstream node that acts to inhibit autophagy. It was found that rapamycin improves MI and inhibits cardiac remodeling by inhibiting mTORC1 and ER stress pathways, preventing angiotensin II-induced apoptosis in h9c2 cells, and promoting autophagy (108).

FUNDC1 was first reported in 2012 as a new hypoxia-induced mitophagy receptor (109). Zhou et al. showed that the pathogenesis of cardiac I/R injury is related to the disruption of mitochondrial homeostasis by CK2α through the inhibition of FUNDC1-related mitophagy. They indicated that CK2α inactivation of FUNDC1, thus effectively inhibiting mitophagy. Defective mitophagy fails to clear damaged mitochondria induced by I/R injury, leading to mitochondrial genome collapse, electron transport chain complex inhibition, mitochondrial biogenesis arrest, mPTP turn-on, and ultimately mitochondrial apoptosis (103). Zhang et al. (110) demonstrated that FUNDC1-mediated mitophagy regulates both mitochondrial quality and quantity *in vivo* under I/R conditions, and plays a role in mitochondrial quality control and functional integrity in platelet activation. Similarly, Mao et al. (111) found that the enhancement of the p-AMPK/FUNDC1 axis can alleviate the hypoxia and reoxygenation induced apoptosis of H9c2 cells and alleviate injury in the I/R mouse model. In addition, Zhang et al. showed that mitophagy in MI is mediated by Rab9-associated autophagosomes, rather than relying on the Atg7 conjugating system and LC3. And they indicated that the Rab9-associated autophagosome pathway is mediated by the synergistic action of Ulk1, RAB9, Rip1, and DRP1 (112).

In conclusion, the role of proteins regulating mitochondrial fusion and fission on MI is divergent, and there seems to be a bidirectional regulation, and the beneficial and detrimental effects still need further validation. Notably, promoting mitophagy is beneficial for MI.

### Cardiac Hypertrophy and Heart Failure

Heart failure (HF) is a syndrome in which the pumping function of the heart is impaired for various reasons and the output of the heart is unable to meet the basic metabolic demands of the tissues of the body (113). It is known that prolonged or high levels of cardiac stress can cause mitochondrial damage and dysfunction in cardiac myocytes. Throughout, mitochondrial metabolism is essential for adequate myocardial pump function, as cardiomyocytes in this state require large amounts of energy to maintain contractile performance, Ca^2+^ homeostasis, and ion transport (114). Based on this by improving mitochondrial function, clearance has the potential to be a strategy for the treatment of HF.

Mitochondrial fusion and fission are associated with myocardial hypertrophy and the development of HF. A study showed that MFN1/MFN2 deficiency-induced eccentric ventricular remodeling with ventricular wall thickening and DRP1 deficiency-induced dilated cardiomyopathy. Further, this study showed that inhibition of the mitochondrial permeability transition alleviates cardiomyocyte death and mitochondrial loss induced by cardiac DRP1 deficiency (115). Another study showed that MFN2 was downregulated in a rat model of myocardial hypertrophy, depending on the etiology and time course of myocardial hypertrophy (116). Therefore, myocardial hypertrophy can be inhibited by up-regulation of MFN2 expression (117, 118). Along similar lines, a clinical study found reduced mitochondrial content, oxidative capacity, and MFN2 expression in skeletal muscle of patients with HF, which is related to the accumulation of dysfunctional organelles and decreased OXPHOS ability in the mitochondrial network due to the low expression of MFN2 (119).

Optic atrophy protein 1 plays an important role in preventing the release of Cytc from the cristae (120). Apoptotic cell death *via* reduction of OPA1 and mitochondrial fusion may contribute to HF progression. Chen et al. (121) found that OPA1 reduction in HF models leads to increased mitochondrial fragmentation, increased apoptosis, increased sensitivity to ischemia. In addition, OPA1 also has a significant effect on energy utilization, OPA1 can increase the utilization of cardiac fatty acids, thereby reducing ROS production and maintaining mitochondrial morphology during HF (122). Two mitochondrial proteases (OMA1 and the AAA protease YME1L) cleave OPA1 from long (L-OPA1) to short (S-OPA1) forms. L-OPA1 is required for mitochondrial fusion, but S-OPA1 is not (123). Wai et al. found that deletion of YME1L activates OMA1, accelerates the proteolytic processing of OPA1, and causes mitochondrial fission of the heart, which changes the metabolism of the heart and further causes dilated cardiomyopathy and HF. However, if deletion of OMA1, OPA1 processing can be avoided and cardiac function and mitochondrial morphology can be restored (124).

Mitophagy in the heart is a homeostatic mechanism for maintaining cardiomyocyte size and global cardiac structure and function, and the upregulation of mitophagy in failing hearts is an adaptive response for protecting cells from hemodynamic stress. For instance, in adult mice, deficiency of Atg5, a protein required for autophagy, led to cardiac hypertrophy, a disorganized sarcomere structure, left ventricular dilatation, and contractile dysfunction, accompanied by increased levels of ubiquitination (125, 126).

Mitophagy is transiently activated and then downregulated in cardiac tissues during the early phase of HF, restoration of mitophagy attenuates dysfunction in the heart during pressure overload. In the presence of pressure overload, treatment with Tat-Beclin 1 can attenuate the progression of HF (127). For instance, a study demonstrated that AMPKα2 protects against the development of HF by enhancing mitophagy *via* PINK1 Phosphorylation (128). Similarly, Beak et al. (129) demonstrated that deficiency of the nuclear receptor RORα in the mouse exacerbates angiotensin II-induced cardiac hypertrophy and compromises cardiomyocyte mitochondrial function, which is associated with reduced mitophagy. Along similar lines, Nah et al. (130) showed that Ulk1-cKO mice had suffered from impaired mitochondrial quality control and more severe cardiac dysfunction, hypertrophy, and myocardial fibrosis, the mechanism of which is related to Ulk1-dependent alternative mitophagy. Lysosomal-associated membrane protein-2 (LAMP-2) is a highly glycosylated protein that is an essential component of the lysosomal membrane. This protein is critical for the fusion of lysosomes and autophagosomes, leading to the degradation of their contents, and LAMP-2 deficiency can lead to premature death in mice and inhibit myocardial systolic function (131). Therefore, overexpression of either autophagy gene and thus increased autophagy levels could improve CMD.

Current evidence overwhelmingly suggests that caloric restriction and fasting induce mitophagy and mitophagy-related markers (132). Fasting or caloric restriction can as a new and practical treatment for CMD. For instance, a study found that caloric restriction can enhance cardiac autophagy and delay cardiac aging by inhibiting the mTOR pathway. Meanwhile, caloric restriction can also reduce the accumulation of senescence-associated β-galactosidase and lipofuscin and reduced myocyte apoptosis (133). Weir et al. (134) found that caloric restriction increased fatty acid oxidation by maintaining mitochondrial network homeostasis and functional coordination with the peroxisome to promote longevity in *C. elegans*. Therefore, it is practical to use non-pharmacological therapies like exercise and diet to treat metabolic diseases.

### Atherosclerosis

Atherosclerosis (AS) is a chronic inflammatory disease of large and medium-sized arteries that causes ischemic heart disease, strokes, and peripheral vascular disease (135). The pathogenesis of AS begins with the accumulation and retention of apolipoprotein B-containing lipoproteins in the vascular sub endothelium which initiates the recruitment and entrance of inflammatory monocytes into the vessel wall. There is increasing evidence that mitochondria play a key role in the development of inflammatory responses and the maintenance of chronic inflammation (136).

Recently, studies have found that age-related mitochondrial dysfunction promotes AS, which is associated with elevated inflammatory factors (137). Mitochondrial damage results in the release of mitochondrial components (mtDNA, cardiolipin), which is a potent DAMP recognized by the immune cells that can trigger the inflammatory response. Exposure to these cellular debris allows the recruitment of adaptor molecules/receptors that triggers an innate immune response (138). It is worth noting that a study has found that mtDNA damage can promote AS independently of ROS through effects on smooth muscle cells and monocytes and correlates with higher-risk plaques in humans (139). Khodzhaeva et al. (140) suggested pro-inflammatory effects of MFN2 deficiency in human macrophages, which could enhance the expression of IL-β and TNF-α, and activate the transduction of the NF-kB signaling pathway. Thus, by correcting mitochondrial dysfunction, it may be possible to reduce the inflammatory state in AS.

It is well-known that mitochondrial generation of ROS is closely related to the occurrence and development of AS. Under normal conditions, mitochondrial antioxidant and repair systems counteract the harmful effects of excess ROS. In addition, mitochondria can counteract the effects of ROS by regulating fusion and fission (141). When mitochondrial dysfunction occurs, ROS is overproduced, leading to the oxidation of lipids, nucleic acids, and proteins, which eventually leads to severe cell damage. Excessive ROS production can lead to endothelial dysfunction, inflammation of blood vessels, and the accumulation of oxLDL in arterial walls (142). By inhibiting DRP-mediated mitochondrial fission, oxidative stress is reduced, endothelial dysfunction is improved, and inflammation is suppressed, thereby reducing the progression of AS (143).

Vascular smooth muscle cells are the main components of the vessel wall and plaques. Abnormal proliferation of VSMCs promotes plaque formation, but VSMCs are beneficial in advanced plaques (preventing rupture of fibrous caps) (144). The pathogenesis of AS is closely related to VSMCs mitochondrial dysfunction (145). DRP1 and mitochondrial fission could promote inflammation and oxidation of VSMCs, and Mdivi1 can significantly inhibit the inflammatory response and oxygen consumption in VSMCs (146). The relationship between MFN2 and AS has been studied for a long time. In 2004, Chen et al. (147) found that MFN2 was significantly reduced in hyperproliferative VSMCs of AS plaques or balloon lesions, and that increasing MFN2 prevented neointimal VSMCs proliferation after balloon injury and prevented restenosis caused by balloon injury. Along this line, another study found that MFN2 constituted a primary determinant of VSMCs apoptosis, and the mechanism is associated with MFN2 mediated Akt signaling pathway as well as a mitochondrial apoptotic pathway, specifically reflected in increasing Bax/Bcl-2 ratio, promoting Cytc release, activating Caspase-9 and Caspase-3 activation (148).

Lack of autophagy genes may severely disrupt intravascular homeostasis (149). Progressive AS has features of dysfunctional autophagy, which is related to plaque macrophages (150). Recently, a study suggested that a high protein diet increases AS progression by activating macrophage mTOR to inhibit mitophagy (151). Macrophages lacking the key autophagic protein ATG5 enhance atherosclerotic plaque formation. Inclusion bodies rich in P62 and polyubiquitinated proteins in macrophages prevent AS (152). Analogously, VSMC-specific ATG7 knockout mice were found to have increased total collagen deposition, nuclear hypertrophy, up-regulation of CDKN2A, reduced phosphorylation of RB, and enhanced GLB1 activity, which is closely related to the promotion of neointima formation and atherosclerosis formation (153). Endothelial autophagy is necessary to maintain vascular lipid homeostasis. By both confocal and electron microscopy, Torisu et al. found that autophagy in endothelial cells may represent an important mechanism for regulating excess exogenous lipids. Their study showed that excess LDL appeared to be engulfed within autophagic structures and that temporary knockdown of ATG7 resulted in increased intracellular levels of I-LDL and oxLDL (154).

Overall, the above evidence demonstrates that mitochondrial dynamic proteins play an important role in inflammation and oxidative stress in AS. Effective mitophagy can play an anti-AS role.

### Obesity

Obesity is a major risk factor for several other diseases, such as diabetes, cardiovascular disease, and metabolic disease. High levels of fat mass accelerate the development of dyslipidemia, blood pressure, inflammation, and glucose/insulin resistance (155, 156). Long-term, high-concentration substrate supplies deplete NAD+, leading to the accumulation of metabolic intermediates, such as acetyl-CoA, which promotes the production of ROS and makes mitochondria vulnerable to damage. Glucose and lipid metabolism are largely dependent on mitochondrial metabolism, and mitochondrial function is most affected in primary tissues, such as bone and heart muscle, liver, and adipose tissue.

It has been found that hyperglycemia and high free fatty acids can modulate insulin sensitivity and lead to mitochondrial dysfunction (157, 158). Several mitochondrial genes critical to mitochondrial function and OXPHOS were down-regulated in obese, HFD fed, insulin-resistant mice (159, 160). Mitochondrial dynamics are influenced by metabolic demands, changes, and alterations. Specific MFN2 mutations lead to tissue-selective mitochondrial dysfunction and increase adipocyte proliferation and survival (161). MFN2 in fat is important for regulating metabolism and has been found to be lower in adipose tissue of mice and obese humans on a HFD (162). Bach et al. showed that weight loss upregulated MFN2 expression in skeletal muscle and type 2 diabetes downregulated MFN2 expression in skeletal muscle in the obese population. Further, MFN2 expression in skeletal muscle was positively associated with insulin sensitivity, but inversely associated with BMI, TNF-α and IL-6 (163). Along this line, Pich et al. (164) found that decreasing MFN2 inhibited the oxidation of pyruvate, glucose and fatty acids and decreased the mitochondrial membrane potential, while increasing MFN2 increased glucose oxidation and mitochondrial membrane potential.

Excessive lipid uptake in the heart affects dynamin and induces mitochondrial fission and dysfunction (165). Lipid overload increased DRP1 level and activity in mouse hearts, and overactivated DRP1 increases mitochondrial fission and interacts with VDAC1 to lead to myocardial cell death, and targeting DRP1 helps reduce cardiac hypertrophy and dysfunction caused by lipid overload. Similarly, it has been reported that blocking DRP-1 on mitochondrial fission can improve white adipose tissue abnormalities in obesity and diabetes in ob/ob mice by short-term leptin and Mdivi-1 treatment (166).

Mitophagy plays an important role in maintaining cardiac function in obesity (167). Defective mitophagy is causally linked to obesity complications. Loss of FUNDC1 aggravates HFD-induced cardiac remodeling, functional and mitochondrial abnormalities, cell death, and Ca^2+^ overload (168). In contrast, a study found that mice lacking muscle FUNDC1 were found to have a protective effect against HFD-induced obesity, with improved insulin sensitivity and glucose tolerance despite reduced muscle mitochondrial energy. The mechanism may be related to FUNDC1 deficiency leading to muscle degeneration and up-regulation of FGF21 expression, thus promoting thermogenic remodeling of adipose tissue (169).

In 2021, Cho et al. reported that serine/threonine-protein kinase 3 (STK3) and STK4 are key physiological suppressors of mitochondrial capacity in brown, beige, and white adipose tissues. They found that STK3 and STK4 increase adipocyte mitophagy in part by regulating the phosphorylation and dimerization status of the mitophagy receptor BNIP3, which increased resistance to metabolic disorders induced by a HFD (170). Increased fatty acid oxidation has been implicated in the development of cardiomyopathy induced by obesity/diabetes. However, Shao D et al. (171) found that increased fatty acid oxidation by regulating Parkin-mediated mitophagy can prevent HFD-induced cardiomyopathy.

## Therapies for Cardiometabolic Diseases

### Mitochondrial Targeted Drug Therapy

Targeting mitochondrial dynamics and mitophagy is increasingly becoming a research direction in the treatment of CMD. DRP1-mediated mitochondrial fission is an attractive drug target. By inhibiting DRP1, excessive mitochondrial fission can be reduced, mitochondrial fusion activity can be enhanced, and loss of mitochondrial membrane potential and release of Cytc can be prevented, thereby preventing cell death (172). Deng et al. (173) found that treatment with Mdivi-1 (20 mg/kg/day) significantly reduced angiotensin II-induced hypertension, arterial remodeling, and cardiac hypertrophy by a mechanism that may be related to preventing phenotypic transformation of VSMCs. Aishwarya et al. (174) found several novel pleiotropy effects of Mdivi-1 in cardiomyocytes, including decreased expression of OXPHOS complex protein and superoxide production, impaired macroautophagy flux, and altered mitochondrial serine protease expression during L-OPA1 proteolysis. Mdivi-1 has been shown to have a cellular protective effect on I/R injury (84, 175). Interestingly, a study found that Mdivi-1 attenuates oxidative stress and exerts vascular protection in I/R injury through a mechanism unrelated to DRP1 activity, possibly due to elevated levels of antioxidant enzymes, SOD1, and catalase, as well as Nrf2 expression (176). Insulin resistance co-occurs with mitochondrial dysfunction in skeletal muscle, and Mdivi-1 can reduce insulin resistance by enhancing mitochondrial function (177, 178).

Melatonin belongs to an indole heterocyclic compound with numerous receptor-mediated and receptor-independent actions It has been found that melatonin can reduce the size of MI, inhibit myocardial cell death, and maintain myocardial function by promoting OPA1-related mitochondrial fusion (179). Further, it was found that melatonin can promote mitochondrial fusion/mitophagy through the AMPK/OPA1 pathway and reduce calcium deposition in VSMCs (180). Zhou et al. (181) showed that melatonin protects cardiac microcirculation from I/R injury by inhibiting mitophagy in PINK1/Parkin pathway and weakening mitochondrial fission. In addition, it was found that melatonin can also protect against diabetic heart disease by inhibiting DRP1 expression through SIRT1-PGC-1α (182). In 2021, Bai et al. (183) demonstrated that melatonin regulates mitophagy and mitochondrial dynamics in a SIRT3-dependent manner to ameliorate hypoxia/reoxygenation damage.

### Mitochondrial Targeted Natural Medicines Therapy

Natural medicines are characterized by multi-target and multi-pathway synergies, and their potential in mitochondrial dynamics is gradually recognized (184). *Panax ginseng* is a traditional herbal medicine that can improve human immunity and ginsenosides are the major bioactive constituents in ginseng root. Yang et al. showed that ginsenoside Rg5 can attenuate I/R injury in cardiomyocytes by inhibiting the opening of mPTP and increasing ATP production. In addition, ginsenoside Rg5 can also inhibit the activation of DRP1 through the AKT pathway, thereby protecting cells from I/R injury (185). Recently, Jiang et al. (186) discovered through proteomics that the mechanism by which ginsenosides Rb1 alleviate I/R injury is that ginsenoside Rb1 reduces the activity of NADH dehydrogenase, making mitochondrial complex I in a deactivated form upon reperfusion, thereby reducing the burst of ROS. Berberine is the extract of *Coptis chinensis* and is widely used. A study showed that berberine improved myocardial hypertrophy and apoptosis induced by transverse aortic contraction in mice by upregulating PINK1/Parkin-mediated mitophagy (187). Gypenosides is an extraction product of *Gynostemma pentaphyllum* (Thunb) Makino. A study found that gypenosides improve AS levels in ApoE–/– mice through the regulation of mitochondrial fission proteins and fusion proteins *via* the PI3K/Akt/Bad pathway (188). Quercetin, widely distributed in the plant kingdom, has a variety of bioactive flavonol compounds. Quercetin can improve vascular calcification (189), insulin sensitivity (190), and I/R injury (191) by inhibiting DRP1-mediated mitochondrial fission. In addition, a study found that quercetin inhibited excessive mitochondrial fission by activating mitophagy *via* SIRT1/TMBIM6, inhibits endoplasmic reticulum stress, and improves human cardiomyocyte activity (192). Related studies on DC also found that quercetin can regulate mitochondrial fusion and fission mechanisms by regulating SIRT5 and IDH2-related succinylation, thereby protecting the pathological damage of myocardial cells stimulated by high glucose (193).

In addition to the active ingredients of traditional Chinese medicine, the compound preparation of traditional Chinese medicine can also have a certain regulatory effect on the interaction mechanism of mitochondrial dynamics and mitophagy. Tongyang Huoxue Recipe, a traditional Chinese medicine compound, can protect sinoatrial node cells under stress through PINK/parkin-mediated mitophagy, and can also inhibit DRP1-mediated mitochondrial fusion/fission dysfunction, increase mitochondrial membrane potential, maintain calcium homeostasis, and inhibits oxidative stress damage (194). Zishen Huoxue Decoction is an effective compound for the treatment of patients with coronary heart disease. Liu et al. (195) found that Zishen Huoxue Decoction has the effects of activating mTORC1 signaling pathway, inhibiting the overexpression of 4E-BP1, inhibiting fatty acid oxidation, protecting the respiratory function of mitochondria, and thus protecting myocardial cells from injury. It is worth noting that natural medicines are subject to many uncertainties and require extensive clinical and basic research for validation.

## Conclusions

Mitochondrial dynamics and mitophagy play an important role in the physiology and pathology of CMD. CMD is closely related to energy metabolism, and effective mitochondrial homeostasis is an important defense mechanism for the heart to deal with various stress and injury. In CMD, mitochondrial dynamics and mitophagy play an important role in improving insulin resistance, improving metabolite utilization, inhibiting inflammatory response, reducing apoptosis, protecting endothelial cells, and reducing ventricular remodeling.

Current evidence overwhelmingly suggested that mitophagy has a positive effect on CMD. Mitochondrial fusion and fission, on the other hand, need to be viewed dialectically. Many studies have shown that MFN1 and MFN2 are beneficial and DRP1 is detrimental, but there are also studies that take the opposite attitude. Therefore, more *in vivo* and *in vitro* studies are still needed for further validation. In terms of targeted mitochondrial therapy, exercise and diet therapy are promising roles without side effects and should be recommended. In addition, natural medicines are a great treasure with great potential from which we can discover better targeted mitochondrial drugs. With the continuous development of modern science and technology and the deepening of research on mitochondrial dynamics and mitophagy drugs, mitochondrial-targeted therapy will provide more ideas for the treatment of CMD, and with the development of new microscopy, biotechnology, and computer technology, the mystery of mitochondria will be gradually explored by human beings.

## Author Contributions

JL and KY designed the manuscript. JL and JD edited the manuscript. SZ, SX, and QW revised the manuscript. All authors contributed to manuscript revision, read, and approved the submitted version.

## Funding

This study was funded by grants from the General Program of National Natural Science Foundation of China (81873173) and the Science and Technology Innovation Project of China Academy of Chinese Medical Sciences (CI2021A00906).

## Conflict of Interest

The authors declare that the research was conducted in the absence of any commercial or financial relationships that could be construed as a potential conflict of interest.

## Publisher's Note

All claims expressed in this article are solely those of the authors and do not necessarily represent those of their affiliated organizations, or those of the publisher, the editors and the reviewers. Any product that may be evaluated in this article, or claim that may be made by its manufacturer, is not guaranteed or endorsed by the publisher.

## References

[1] Mechanick JI Farkouh ME Newman JD Garvey WT. Cardiometabolic-based chronic disease, addressing knowledge and clinical practice gaps: JACC state-of-the-art review. J Am Coll Cardiol. (2020) 75:539–55. 10.1016/j.jacc.2019.11.046 32029137 PMC8168371

[2] Mechanick JI Farkouh ME Newman JD Garvey WT. Cardiometabolic-based chronic disease, adiposity and dysglycemia drivers: JACC state-of-the-art review. J Am Coll Cardiol. (2020) 75:525–38. 10.1016/j.jacc.2019.11.044 32029136 PMC7187687

[3] Roth GA Mensah GA Johnson CO Addolorato G Ammirati E Baddour LM. Global burden of cardiovascular diseases and risk factors, 1990–2019: update from the GBD 2019 study. J Am Coll Cardiol. (2020) 76:2982–3021. 10.1016/j.jacc.2020.11.010 33309175 PMC7755038

[4] Bonora M Wieckowski MR Sinclair DA Kroemer G Pinton P Galluzzi L. Targeting mitochondria for cardiovascular disorders: therapeutic potential and obstacles. Nat Rev Cardiol. (2019) 16:33–55. 10.1038/s41569-018-0074-0 30177752 PMC6349394

[5] Honka H Solis-Herrera C Triplitt C Norton L Butler J DeFronzo RA. Therapeutic manipulation of myocardial metabolism: JACC state-of-the-art review. J Am Coll Cardiol. (2021) 77:2022–39. 10.1016/j.jacc.2021.02.057 33888253 PMC8091273

[6] Lopaschuk GD Karwi QG Tian R Wende AR Abel ED. Cardiac energy metabolism in heart failure. Circ Res. (2021) 128:1487–513. 10.1161/CIRCRESAHA.121.318241 33983836 PMC8136750

[7] Lopaschuk GD Ussher JR Folmes CD Jaswal JS Stanley WC. Myocardial fatty acid metabolism in health and disease. Physiol Rev. (2010) 90:207–58. 10.1152/physrev.00015.2009 20086077

[8] Smith RL Soeters MR Wüst RCI Houtkooper RH. Metabolic flexibility as an adaptation to energy resources and requirements in health and disease. Endocr Rev. (2018) 39:489–517. 10.1210/er.2017-00211 29697773 PMC6093334

[9] Makrecka-Kuka M Liepinsh E Murray AJ Lemieux H Dambrova M Tepp K. Altered mitochondrial metabolism in the insulin-resistant heart. Acta Physiologica. (2020) 228:e13430. 10.1111/apha.13430 31840389

[10] Deng Y Xie M Li Q Xu X Ou W Zhang Y. Targeting mitochondria-inflammation circuit by B-Hydroxybutyrate mitigates Hfpef. Circ Res. (2021) 128:232–45. 10.1161/CIRCRESAHA.120.317933 33176578

[11] Gibb AA Hill BG. Metabolic co-ordination of physiological and pathological cardiac remodeling. Circ Res. (2018) 123:107–28. 10.1161/CIRCRESAHA.118.312017 29929976 PMC6023588

[12] Roger AJ Muñoz-Gómez SA Kamikawa R. The origin and diversification of mitochondria. Curr Biol. (2017) 27:R1177–92. 10.1016/j.cub.2017.09.015 29112874

[13] Tahrir FG Langford D Amini S Mohseni Ahooyi T Khalili K. Mitochondrial quality control in cardiac cells: mechanisms and role in cardiac cell injury and disease. J Cell Physiol. (2019) 234:8122–33. 10.1002/jcp.27597 30417391 PMC6395499

[14] Ng MYW Wai T Simonsen A. Quality control of the mitochondrion. Dev Cell. (2021) 56:881–905. 10.1016/j.devcel.2021.02.009 33662258

[15] Zhang D Li Y Heims-Waldron D Bezzerides V Guatimosim S Guo Y. Mitochondrial cardiomyopathy caused by elevated reactive oxygen species and impaired cardiomyocyte proliferation. Circ Res. (2018) 122:74–87. 10.1161/CIRCRESAHA.117.311349 29021295 PMC5756124

[16] Dia M Gomez L Thibault H Tessier N Leon C Chouabe C. Reduced reticulum-mitochondria Ca (2+) transfer is an early and reversible trigger of mitochondrial dysfunctions in diabetic cardiomyopathy. Basic Res Cardiol. (2020) 115:74. 10.1007/s00395-020-00835-7 33258101 PMC7704523

[17] Hanif Sayyed UM Mahalakshmi R. Mitochondrial protein translocation machinery: from tom structural biogenesis to functional regulation. J Biol Chem. (2022) 298:101870. 10.1016/j.jbc.2022.101870 35346689 PMC9052162

[18] Araiso Y Tsutsumi A Qiu J Imai K Shiota T Song J. Structure of the mitochondrial import gate reveals distinct pre-protein paths. Nature. (2019) 575:395–401. 10.1038/s41586-019-1680-7 31600774

[19] Pape JK Stephan T Balzarotti F Büchner R Lange F Riedel D. Multicolor 3d minflux nanoscopy of mitochondrial micos proteins. Proc Natl Acad Sci USA. (2020) 117:20607–14. 10.1073/pnas.2009364117 32788360 PMC7456099

[20] Gilkerson RW Selker JM Capaldi RA. The cristal membrane of mitochondria is the principal site of oxidative phosphorylation. FEBS Lett. (2003) 546:355–8. 10.1016/S0014-5793(03)00633-1 12832068

[21] Klecker T Westermann B. Pathways shaping the mitochondrial inner membrane. Open Biol. (2021) 11:210238. 10.1098/rsob.210238 34847778 PMC8633786

[22] Becker T Gebert M Pfanner N van der Laan M. Biogenesis of mitochondrial membrane proteins. Curr Opin Cell Biol. (2009) 21:484–93. 10.1016/j.ceb.2009.04.002 19423316

[23] Protasoni M Zeviani M. Mitochondrial structure and bioenergetics in normal and disease conditions. Int J Mol Sci. (2021) 22:586. 10.3390/ijms22020586 33435522 PMC7827222

[24] Kaasik A Safiulina D Zharkovsky A Veksler V. Regulation of mitochondrial matrix volume. Am J Physiol Cell Physiol. (2007) 292:C157–63. 10.1152/ajpcell.00272.2006 16870828

[25] Yan C Duanmu X Zeng L Liu B Song Z. Mitochondrial DNA: distribution, mutations, and elimination. Cells. (2019) 8:379. 10.3390/cells8040379 31027297 PMC6523345

[26] Sharma P Sampath H. Mitochondrial DNA integrity: role in health and disease. Cells. (2019) 8:100. 10.3390/cells8020100 30700008 PMC6406942

[27] Chen Q Sun L Chen ZJ. Regulation and function of the Cgas-sting pathway of cytosolic DNA sensing. Nat Immunol. (2016) 17:1142–9. 10.1038/ni.3558 27648547

[28] Youle RJ van der Bliek AM. Mitochondrial fission, fusion, and stress. Science. (2012) 337:1062–5. 10.1126/science.1219855 22936770 PMC4762028

[29] Tilokani L Nagashima S Paupe V Prudent J. Mitochondrial dynamics: overview of molecular mechanisms. Essays Biochem. (2018) 62:341–60. 10.1042/EBC20170104 30030364 PMC6056715

[30] Szabo A Sumegi K Fekete K Hocsak E Debreceni B Setalo G Jr. Activation of mitochondrial fusion provides a new treatment for mitochondria-related diseases. Biochem Pharmacol. (2018) 150:86–96. 10.1016/j.bcp.2018.01.038 29378182

[31] Yoneda M Miyatake T Attardi G. Complementation of mutant and wild-type human mitochondrial DNAs co-existing since the mutation event and lack of complementation of DNAs introduced separately into a cell within distinct organelles. Mol Cell Biol. (1994) 14:2699–712. 10.1128/mcb.14.4.2699-2712.1994 8139569 PMC358636

[32] Taguchi N Ishihara N Jofuku A Oka T Mihara K. Mitotic phosphorylation of dynamin-related Gtpase Drp1 participates in mitochondrial fission. J Biol Chem. (2007) 282:11521–9. 10.1074/jbc.M607279200 17301055

[33] Zheng Z Xiang S Wang Y Dong Y Li Z Xiang Y. Nr4a1 promotes Tnf-A-induced chondrocyte death and migration injury *via* activating the ampk/Drp1/mitochondrial fission pathway. Int J Mol Med. (2020) 45:151–61. 10.3892/ijmm.2019.4398 31746366 PMC6889925

[34] Twig G Elorza A Molina AJ Mohamed H Wikstrom JD Walzer G. Fission and selective fusion govern mitochondrial segregation and elimination by autophagy. EMBO J. (2008) 27:433–46. 10.1038/sj.emboj.7601963 18200046 PMC2234339

[35] Song M Franco A Fleischer JA Zhang L Dorn GW 2nd. Abrogating mitochondrial dynamics in mouse hearts accelerates mitochondrial senescence. Cell Metab. (2017) 26:872–83.e5. 10.1016/j.cmet.2017.09.023 29107503 PMC5718956

[36] Ferguson SM De Camilli P. Dynamin, a membrane-remodelling Gtpase. Nat Rev Mol Cell Biol. (2012) 13:75–88. 10.1038/nrm3266 22233676 PMC3519936

[37] Cipolat S Martins de Brito O Dal Zilio B Scorrano L. Opa1 requires Mitofusin 1 to promote mitochondrial fusion. Proc Natl Acad Sci USA. (2004) 101:15927–32. 10.1073/pnas.0407043101 15509649 PMC528769

[38] Gao S Hu J. Mitochondrial fusion: the machineries in and out. Trends Cell Biol. (2021) 31:62–74. 10.1016/j.tcb.2020.09.008 33092941

[39] Chandhok G Lazarou M Neumann B. Structure, function, and regulation of Mitofusin-2 in health and disease. Biol Rev Camb Philos Soc. (2018) 93:933–49. 10.1111/brv.12378 29068134 PMC6446723

[40] Chen H Detmer SA Ewald AJ Griffin EE Fraser SE Chan DC. Mitofusins Mfn1 and Mfn2 co-ordinately regulate mitochondrial fusion and are essential for embryonic development. J Cell Biol. (2003) 160:189–200. 10.1083/jcb.200211046 12527753 PMC2172648

[41] Giacomello M Pyakurel A Glytsou C Scorrano L. The cell biology of mitochondrial membrane dynamics. Nat Rev Mol Cell Biol. (2020) 21:204–24. 10.1038/s41580-020-0210-7 32071438

[42] Palmer CS Osellame LD Laine D Koutsopoulos OS Frazier AE Ryan MT. Mid49 and Mid51, new components of the mitochondrial fission machinery. EMBO Rep. (2011) 12:565–73. 10.1038/embor.2011.54 21508961 PMC3128275

[43] Lee JE Westrate LM Wu H Page C Voeltz GK. Multiple dynamin family members collaborate to drive mitochondrial division. Nature. (2016) 540:139–43. 10.1038/nature20555 27798601 PMC5656044

[44] Koch A Thiemann M Grabenbauer M Yoon Y McNiven MA Schrader M. Dynamin-like protein 1 is involved in peroxisomal fission. J Biol Chem. (2003) 278:8597–605. 10.1074/jbc.M211761200 12499366

[45] Kageyama Y Hoshijima M Seo K Bedja D Sysa-Shah P Andrabi SA. Parkin-independent mitophagy requires Drp1 and maintains the integrity of mammalian heart and brain. EMBO J. (2014) 33:2798–813. 10.15252/embj.201488658 25349190 PMC4282557

[46] Dorn GW 2nd Vega RB Kelly DP. Mitochondrial biogenesis and dynamics in the developing and diseased heart. Genes Dev. (2015) 29:1981–91. 10.1101/gad.269894.115 26443844 PMC4604339

[47] Lee TW Bai KJ Lee TI Chao TF Kao YH Chen YJ. Ppars modulate cardiac metabolism and mitochondrial function in diabetes. J Biomed Sci. (2017) 24:5. 10.1186/s12929-016-0309-5 28069019 PMC5223385

[48] Del Re DP Amgalan D Linkermann A Liu Q Kitsis RN. Fundamental mechanisms of regulated cell death and implications for heart disease. Physiol Rev. (2019) 99:1765–817. 10.1152/physrev.00022.2018 31364924 PMC6890986

[49] Gupta S Kass GE Szegezdi E Joseph B. The mitochondrial death pathway: a promising therapeutic target in diseases. J Cell Mol Med. (2009) 13:1004–33. 10.1111/j.1582-4934.2009.00697.x 19220575 PMC4496101

[50] Jornayvaz FR Shulman GI. Regulation of mitochondrial biogenesis. Essays Biochem. (2010) 47:69–84. 10.1042/bse0470069 20533901 PMC3883043

[51] Fang WJ Wang CJ He Y Zhou YL Peng XD Liu SK. Resveratrol alleviates diabetic cardiomyopathy in rats by improving mitochondrial function through Pgc-1α deacetylation. Acta Pharmacol Sin. (2018) 39:59–73. 10.1038/aps.2017.50 28770830 PMC5758665

[52] Haemmerle G Moustafa T Woelkart G Büttner S Schmidt A van de Weijer T. Atgl-mediated fat catabolism regulates cardiac mitochondrial function *via* Ppar-A and Pgc-1. Nat Med. (2011) 17:1076–85. 10.1038/nm.2439 21857651 PMC3244833

[53] Martin OJ Lai L Soundarapandian MM Leone TC Zorzano A Keller MP. A role for peroxisome proliferator-activated receptor Γ co-activator-1 in the control of mitochondrial dynamics during postnatal cardiac growth. Circ Res. (2014) 114:626–36. 10.1161/CIRCRESAHA.114.302562 24366168 PMC4061768

[54] Lai L Leone TC Zechner C Schaeffer PJ Kelly SM Flanagan DP. Transcriptional co-activators Pgc-1alpha and Pgc-Lbeta control overlapping programs required for perinatal maturation of the heart. Genes Dev. (2008) 22:1948–61. 10.1101/gad.1661708 18628400 PMC2492740

[55] Dikic I Elazar Z. Mechanism and medical implications of mammalian autophagy. Nat Rev Mol Cell Biol. (2018) 19:349–64. 10.1038/s41580-018-0003-4 29618831

[56] Galluzzi L Pietrocola F Levine B Kroemer G. Metabolic control of autophagy. Cell. (2014) 159:1263–76. 10.1016/j.cell.2014.11.006 25480292 PMC4500936

[57] Lavandero S Chiong M Rothermel BA Hill JA. Autophagy in cardiovascular biology. J Clin Invest. (2015) 125:55–64. 10.1172/JCI73943 25654551 PMC4382263

[58] Dewanjee S Vallamkondu J Kalra RS John A Reddy PH Kandimalla R. Autophagy in the diabetic heart: a potential pharmacotherapeutic target in diabetic cardiomyopathy. Ageing Res Rev. (2021) 68:101338. 10.1016/j.arr.2021.101338 33838320

[59] Bravo-San Pedro JM Kroemer G Galluzzi L. Autophagy and mitophagy in cardiovascular disease. Circ Res. (2017) 120:1812–24. 10.1161/CIRCRESAHA.117.311082 28546358

[60] Morales PE Arias-Durán C Ávalos-Guajardo Y Aedo G Verdejo HE Parra V. Emerging role of mitophagy in cardiovascular physiology and pathology. Mol Aspects Med. (2020) 71:100822. 10.1016/j.mam.2019.09.006 31587811

[61] Shires SE Gustafsson Å B. Mitophagy and heart failure. J Mol Med. (2015) 93:253–62. 10.1007/s00109-015-1254-6 25609139 PMC4334711

[62] Tong M Saito T Zhai P Oka SI Mizushima W Nakamura M. Mitophagy is essential for maintaining cardiac function during high fat diet-induced diabetic cardiomyopathy. Circ Res. (2019) 124:1360–71. 10.1161/CIRCRESAHA.118.314607 30786833 PMC6483841

[63] Zhao W Li Y Jia L Pan L Li H Du J. Atg5 deficiency-mediated mitophagy aggravates cardiac inflammation and injury in response to angiotensin II. Free Radic Biol Med. (2014) 69:108–15. 10.1016/j.freeradbiomed.2014.01.002 24418158

[64] Dillmann WH. Diabetic cardiomyopathy. Circ Res. (2019) 124:1160–2. 10.1161/CIRCRESAHA.118.314665 30973809 PMC6578576

[65] Gollmer J Zirlik A Bugger H. Mitochondrial mechanisms in diabetic cardiomyopathy. Diabetes Metab J. (2020) 44:33–53. 10.4093/dmj.2019.0185 32097997 PMC7043970

[66] Sung MM Hamza SM Dyck JR. Myocardial metabolism in diabetic cardiomyopathy: potential therapeutic targets. Antioxid Redox Signal. (2015) 22:1606–30. 10.1089/ars.2015.6305 25808033

[67] Liesa M Shirihai OS. Mitochondrial dynamics in the regulation of nutrient utilization and energy expenditure. Cell Metab. (2013) 17:491–506. 10.1016/j.cmet.2013.03.002 23562075 PMC5967396

[68] Yu T Robotham JL Yoon Y. Increased production of reactive oxygen species in hyperglycemic conditions requires dynamic change of mitochondrial morphology. Proc Natl Acad Sci USA. (2006) 103:2653–8. 10.1073/pnas.0511154103 16477035 PMC1413838

[69] Hu L Ding M Tang D Gao E Li C Wang K. Targeting mitochondrial dynamics by regulating Mfn2 for therapeutic intervention in diabetic cardiomyopathy. Theranostics. (2019) 9:3687–706. 10.7150/thno.33684 31281507 PMC6587356

[70] Sebastián D Hernández-Alvarez MI Segalés J Sorianello E Muñoz JP Sala D. Mitofusin 2 (Mfn2) links mitochondrial and endoplasmic reticulum function with insulin signaling and is essential for normal glucose homeostasis. Proc Natl Acad Sci USA. (2012) 109:5523–8. 10.1073/pnas.1108220109 22427360 PMC3325712

[71] Montaigne D Marechal X Coisne A Debry N Modine T Fayad G. Myocardial contractile dysfunction is associated with impaired mitochondrial function and dynamics in type 2 diabetic but not in obese patients. Circulation. (2014) 130:554–64. 10.1161/CIRCULATIONAHA.113.008476 24928681

[72] Zheng H Zhu H Liu X Huang X Huang A Huang Y. Mitophagy in diabetic cardiomyopathy: roles and mechanisms. Front Cell Dev Biol. (2021) 9:750382. 10.3389/fcell.2021.750382 34646830 PMC8503602

[73] Mu J Zhang D Tian Y Xie Z Zou MH. Brd4 inhibition by Jq1 prevents high-fat diet-induced diabetic cardiomyopathy by activating Pink1/Parkin-mediated mitophagy *in vivo*. J Mol Cell Cardiol. (2020) 149:1–14. 10.1016/j.yjmcc.2020.09.003 32941882 PMC7736123

[74] Wu H Wang Y Li W Chen H Du L Liu D. Deficiency of mitophagy receptor Fundc1 impairs mitochondrial quality and aggravates dietary-induced obesity and metabolic syndrome. Autophagy. (2019) 15:1882–98. 10.1080/15548627.2019.1596482 30898010 PMC6844496

[75] Donath MY Dinarello CA Mandrup-Poulsen T. Targeting innate immune mediators in type 1 and type 2 diabetes. Nat Rev Immunol. (2019) 19:734–46. 10.1038/s41577-019-0213-9 31501536

[76] Chen J Stimpson SE Fernandez-Bueno GA Mathews CE. Mitochondrial reactive oxygen species and type 1 diabetes. Antioxid Redox Signal. (2018) 29:1361–72. 10.1089/ars.2017.7346 29295631 PMC6166689

[77] He F Huang Y Song Z Zhou HJ Zhang H Perry RJ. Mitophagy-mediated adipose inflammation contributes to type 2 diabetes with hepatic insulin resistance. J Exp Med. (2021) 218:e20201416. 10.1084/jem.20201416 33315085 PMC7927432

[78] Sidarala V Pearson GL Parekh VS Thompson B Christen L Gingerich MA. Mitophagy protects B cells from inflammatory damage in diabetes. JCI Insight. (2020) 5:e141138. 10.1172/jci.insight.141138 33232298 PMC7819751

[79] Tanajak P Pintana H Siri-Angkul N Khamseekaew J Apaijai N Chattipakorn SC. Vildagliptin and caloric restriction for cardioprotection in pre-diabetic rats. J Endocrinol. (2017) 232:189–204. 10.1530/JOE-16-0406 27875248

[80] Fealy CE Mulya A Lai N Kirwan JP. Exercise training decreases activation of the mitochondrial fission protein dynamin-related protein-1 in insulin-resistant human skeletal muscle. J Appl Physiol. (2014) 117:239–45. 10.1152/japplphysiol.01064.2013 24947026 PMC4122691

[81] Thygesen K Alpert JS Jaffe AS Chaitman BR Bax JJ Morrow DA. Fourth universal definition of myocardial infarction (2018). J Am Coll Cardiol. (2018) 72:2231–64. 10.1016/j.jacc.2018.08.1038 30153967

[82] Hausenloy DJ Yellon DM. Myocardial ischemia-reperfusion injury: a neglected therapeutic target. J Clin Invest. (2013) 123:92–100. 10.1172/JCI62874 23281415 PMC3533275

[83] Ramachandra CJA Hernandez-Resendiz S Crespo-Avilan GE Lin YH Hausenloy DJ. Mitochondria in acute myocardial infarction and cardioprotection. EBio Med. (2020) 57:102884. 10.1016/j.ebiom.2020.102884 32653860 PMC7355051

[84] Ong SB Subrayan S Lim SY Yellon DM Davidson SM Hausenloy DJ. Inhibiting mitochondrial fission protects the heart against ischemia/reperfusion injury. Circulation. (2010) 121:2012–22. 10.1161/CIRCULATIONAHA.109.906610 20421521

[85] Ding M Dong Q Liu Z Liu Z Qu Y Li X. Inhibition of dynamin-related protein 1 protects against myocardial ischemia-reperfusion injury in diabetic mice. Cardiovasc Diabetol. (2017) 16:19. 10.1186/s12933-017-0501-2 28173848 PMC5297196

[86] Liu J Yan W Zhao X Jia Q Wang J Zhang H. Sirt3 attenuates post-infarction cardiac injury *via* inhibiting mitochondrial fission and normalization of Ampk-Drp1 pathways. Cell Signal. (2019) 53:1–13. 10.1016/j.cellsig.2018.09.009 30219671

[87] Ikeda Y Shirakabe A Maejima Y Zhai P Sciarretta S Toli J. Endogenous Drp1 mediates mitochondrial autophagy and protects the heart against energy stress. Circ Res. (2015) 116:264–78. 10.1161/CIRCRESAHA.116.303356 25332205

[88] Maneechote C Palee S Kerdphoo S Jaiwongkam T Chattipakorn SC Chattipakorn N. Differential temporal inhibition of mitochondrial fission by Mdivi-1 exerts effective cardioprotection in cardiac ischemia/reperfusion injury. Clin Sci. (2018) 132:1669–83. 10.1042/CS20180510 30065084

[89] Ong SB Kwek XY Katwadi K Hernandez-Resendiz S Crespo-Avilan GE Ismail NI. Targeting mitochondrial fission using Mdivi-1 in a clinically relevant large animal model of acute myocardial infarction: a pilot study. Int J Mol Sci. (2019) 20:3972. 10.3390/ijms20163972 31443187 PMC6720595

[90] Zhang J Wang L Xie W Hu S Zhou H Zhu P. Melatonin attenuates Er stress and mitochondrial damage in septic cardiomyopathy: a new mechanism involving Bap31 upregulation and Mapk-Erk pathway. J Cell Physiol. (2020) 235:2847–56. 10.1002/jcp.29190 31535369

[91] Cheng D Zheng J Hu F Lv W Lu C. Abnormal mitochondria-endoplasmic reticulum communication promotes myocardial infarction. Front Physiol. (2021) 12:717187. 10.3389/fphys.2021.717187 34413791 PMC8369510

[92] Wang J Zhou H. Mitochondrial quality control mechanisms as molecular targets in cardiac ischemia-reperfusion injury. Acta Pharm Sin B. (2020) 10:1866–79. 10.1016/j.apsb.2020.03.004 33163341 PMC7606115

[93] Dai SH Wu QC Zhu RR Wan XM Zhou XL. Notch1 protects against myocardial ischaemia-reperfusion injury *via* regulating mitochondrial fusion and function. J Cell Mol Med. (2020) 24:3183–91. 10.1111/jcmm.14992 31975567 PMC7077547

[94] Liu M Li X Huang D. Mfn2 overexpression attenuates cardio-cerebrovascular ischemia-reperfusion injury through mitochondrial fusion and activation of the Ampk/Sirt3 signaling. Front Cell Dev Biol. (2020) 8:598078. 10.3389/fcell.2020.598078 33195281 PMC7644524

[95] Hall AR Burke N Dongworth RK Kalkhoran SB Dyson A Vicencio JM. Hearts deficient in both Mfn1 and Mfn2 are protected against acute myocardial infarction. Cell Death Dis. (2016) 7:e2238. 10.1038/cddis.2016.139 27228353 PMC4917668

[96] Papanicolaou KN Ngoh GA Dabkowski ER O'Connell KA Ribeiro RF Jr Stanley WC. Cardiomyocyte deletion of mitofusin-1 leads to mitochondrial fragmentation and improves tolerance to Ros-induced mitochondrial dysfunction and cell death. Am J Physiol - Heart Circ Physiol. (2012) 302:H167–79. 10.1152/ajpheart.00833.2011 22037195 PMC3334239

[97] Wang M Wang RY Zhou JH Xie XH Sun GB Sun XB. Calenduloside E ameliorates myocardial ischemia-reperfusion injury through regulation of Ampk and mitochondrial Opa1. Oxid Med Cell Longev. (2020) 2020:2415269. 10.1155/2020/2415269 32934760 PMC7479459

[98] Guan L Che Z Meng X Yu Y Li M Yu Z. Mcu up-regulation contributes to myocardial ischemia-reperfusion injury through Calpain/Opa-1-mediated mitochondrial fusion/mitophagy inhibition. J Cell Mol Med. (2019) 23:7830–43. 10.1111/jcmm.14662 31502361 PMC6815825

[99] Le Page S Niro M Fauconnier J Cellier L Tamareille S Gharib A. Increase in cardiac ischemia-reperfusion injuries in Opa1+/- mouse model. PLoS ONE. (2016) 11:e0164066. 10.1371/journal.pone.0164066 27723783 PMC5056696

[100] Zhang Y Wang Y Xu J Tian F Hu S Chen Y. Melatonin attenuates myocardial ischemia-reperfusion injury *via* improving mitochondrial fusion/mitophagy and activating the Ampk-Opa1 signaling pathways. J Pineal Res. (2019) 66:e12542. 10.1111/jpi.12542 30516280

[101] Li L Tan J Miao Y Lei P Zhang Q. Ros and autophagy: interactions and molecular regulatory mechanisms. Cell Mol Neurobiol. (2015) 35:615–21. 10.1007/s10571-015-0166-x 25722131 PMC11486209

[102] Cai Y Yang E Yao X Zhang X Wang Q Wang Y. Fundc1-dependent mitophagy induced by Tpa protects neurons against cerebral ischemia-reperfusion injury. Redox Biol. (2021) 38:101792. 10.1016/j.redox.2020.101792 33212415 PMC7679257

[103] Zhou H Zhu P Wang J Zhu H Ren J Chen Y. Pathogenesis of cardiac ischemia reperfusion injury is associated with Ck2α-disturbed mitochondrial homeostasis *via* suppression of Fundc1-related mitophagy. Cell Death Differ. (2018) 25:1080–93. 10.1038/s41418-018-0086-7 29540794 PMC5988750

[104] Gouspillou G Godin R Piquereau J Picard M Mofarrahi M Mathew J. Protective role of parkin in skeletal muscle contractile and mitochondrial function. J Physiol. (2018) 596:2565–79. 10.1113/JP275604 29682760 PMC6023825

[105] Kubli DA Zhang X Lee Y Hanna RA Quinsay MN Nguyen CK. Parkin protein deficiency exacerbates cardiac injury and reduces survival following myocardial infarction. J Biol Chem. (2013) 288:915–26. 10.1074/jbc.M112.411363 23152496 PMC3543040

[106] Siddall HK Yellon DM Ong SB Mukherjee UA Burke N Hall AR. Loss of Pink1 increases the heart's vulnerability to ischemia-reperfusion injury. PLoS ONE. (2013) 8:e62400. 10.1371/journal.pone.0062400 23638067 PMC3639249

[107] Liu W Chen C Gu X Zhang L Mao X Chen Z. Am1241 alleviates myocardial ischemia-reperfusion injury in rats by enhancing Pink1/Parkin-mediated autophagy. Life Sci. (2021) 272:119228. 10.1016/j.lfs.2021.119228 33607150

[108] Gao G Chen W Yan M Liu J Luo H Wang C. Rapamycin regulates the balance between cardiomyocyte apoptosis and autophagy in chronic heart failure by inhibiting Mtor signaling. Int J Mol Med. (2020) 45:195–209. 10.3892/ijmm.2019.4407 31746373 PMC6889932

[109] Liu L Feng D Chen G Chen M Zheng Q Song P. Mitochondrial outer-membrane protein Fundc1 mediates hypoxia-induced mitophagy in mammalian cells. Nat Cell Biol. (2012) 14:177–85. 10.1038/ncb2422 22267086

[110] Zhang W Siraj S Zhang R Chen Q. Mitophagy receptor Fundc1 regulates mitochondrial homeostasis and protects the heart from I/R injury. Autophagy. (2017) 13:1080–1. 10.1080/15548627.2017.1300224 28323531 PMC5486361

[111] Mao S Tian S Luo X Zhou M Cao Z Li J. Overexpression of Plk1 relieved the myocardial ischemia-reperfusion injury of rats through inducing the mitophagy and regulating the P-Ampk/Fundc1 axis. Bioengineered. (2021) 12:2676–87. 10.1080/21655979.2021.1938500 34115550 PMC8806532

[112] Saito T Nah J Oka SI Mukai R Monden Y Maejima Y. An alternative mitophagy pathway mediated by Rab9 protects the heart against ischemia. J Clin Invest. (2019) 129:802–19. 10.1172/JCI122035 30511961 PMC6355232

[113] Baman JR Ahmad FS. Heart failure. JAMA. (2020) 324:1015. 10.1001/jama.2020.13310 32749448

[114] Huss JM Kelly DP. Mitochondrial energy metabolism in heart failure: a question of balance. J Clin Invest. (2005) 115:547–55. 10.1172/JCI24405 15765136 PMC1052011

[115] Song M Mihara K Chen Y Scorrano L Dorn GW 2nd. Mitochondrial fission and fusion factors reciprocally orchestrate mitophagic culling in mouse hearts and cultured fibroblasts. Cell Metab. (2015) 21:273–86. 10.1016/j.cmet.2014.12.011 25600785 PMC4318753

[116] Fang L Moore XL Gao XM Dart AM Lim YL Du XJ. Down-regulation of Mitofusin-2 expression in cardiac hypertrophy *in vitro* and *in vivo*. Life Sci. (2007) 80:2154–60. 10.1016/j.lfs.2007.04.003 17499311

[117] Yu H Guo Y Mi L Wang X Li L Gao W. Mitofusin 2 inhibits angiotensin II-induced myocardial hypertrophy. J Cardiovasc Pharmacol Ther. (2011) 16:205–11. 10.1177/1074248410385683 21106870

[118] Sun D Li C Liu J Wang Z Liu Y Luo C. Expression profile of micro RNAs in hypertrophic cardiomyopathy and effects of micro RNA-20 in inducing cardiomyocyte hypertrophy through regulating gene Mfn2. DNA Cell Biol. (2019) 38:796–807. 10.1089/dna.2019.4731 31295012

[119] Molina AJ Bharadwaj MS Van Horn C Nicklas BJ Lyles MF Eggebeen J. Skeletal muscle mitochondrial content, oxidative capacity, and Mfn2 expression are reduced in older patients with heart failure and preserved ejection fraction and are related to exercise intolerance. JACC Heart Fail. (2016) 4:636–45. 10.1016/j.jchf.2016.03.011 27179829 PMC4967040

[120] Frezza C Cipolat S Martins de Brito O Micaroni M Beznoussenko GV Rudka T. Opa1 controls apoptotic cristae remodeling independently from mitochondrial fusion. Cell. (2006) 126:177–89. 10.1016/j.cell.2006.06.025 16839885

[121] Chen L Gong Q Stice JP Knowlton AA. Mitochondrial Opa1, apoptosis, and heart failure. Cardiovasc Res. (2009) 84:91–9. 10.1093/cvr/cvp181 19493956 PMC2741347

[122] Guo Y Wang Z Qin X Xu J Hou Z Yang H. Enhancing fatty acid utilization ameliorates mitochondrial fragmentation and cardiac dysfunction *via* rebalancing optic atrophy 1 processing in the failing heart. Cardiovasc Res. (2018) 114:979–91. 10.1093/cvr/cvy052 29490017

[123] Anand R Wai T Baker MJ Kladt N Schauss AC Rugarli E. The I-Aaa protease Yme1l and Oma1 cleave Opa1 to balance mitochondrial fusion and fission. J Cell Biol. (2014) 204:919–29. 10.1083/jcb.201308006 24616225 PMC3998800

[124] Wai T García-Prieto J Baker MJ Merkwirth C Benit P Rustin P. Imbalanced Opa1 processing and mitochondrial fragmentation cause heart failure in mice. Science. (2015) 350:aad0116. 10.1126/science.aad0116 26785494

[125] Nakai A Yamaguchi O Takeda T Higuchi Y Hikoso S Taniike M. The role of autophagy in cardiomyocytes in the basal state and in response to hemodynamic stress. Nat Med. (2007) 13:619–24. 10.1038/nm1574 17450150

[126] Taneike M Yamaguchi O Nakai A Hikoso S Takeda T Mizote I. Inhibition of autophagy in the heart induces age-related cardiomyopathy. Autophagy. (2010) 6:600–6. 10.4161/auto.6.5.11947 20431347

[127] Shirakabe A Zhai P Ikeda Y Saito T Maejima Y Hsu CP. Drp1-dependent mitochondrial autophagy plays a protective role against pressure overload-induced mitochondrial dysfunction and heart failure. Circulation. (2016) 133:1249–63. 10.1161/CIRCULATIONAHA.115.020502 26915633 PMC4811679

[128] Wang B Nie J Wu L Hu Y Wen Z Dong L. Ampkα2 protects against the development of heart failure by enhancing mitophagy *via* Pink1 phosphorylation. Circ Res. (2018) 122:712–29. 10.1161/CIRCRESAHA.117.312317 29284690 PMC5834386

[129] Beak JY Kang HS Huang W Deshmukh R Hong SJ Kadakia N. The nuclear receptor Rorα preserves cardiomyocyte mitochondrial function by regulating caveolin-3-mediated mitophagy. J Biol Chem. (2021) 297:101358. 10.1016/j.jbc.2021.101358 34756888 PMC8626585

[130] Nah J Shirakabe A Mukai R Zhai P Sung EA Ivessa A. Ulk1-dependent alternative mitophagy plays a protective role during pressure overload in the heart. Cardiovasc Res. (2022). 10.1093/cvr/cvac003. [Epub ahead of print]. 35018428 PMC10144728

[131] Stypmann J Janssen PM Prestle J Engelen MA Kögler H Lüllmann-Rauch R. Lamp-2 deficient mice show depressed cardiac contractile function without significant changes in calcium handling. Basic Res Cardiol. (2006) 101:281–91. 10.1007/s00395-006-0591-6 16604439

[132] Mehrabani S Bagherniya M Askari G Read MI Sahebkar A. The effect of fasting or calorie restriction on mitophagy induction: a literature review. J Cachexia Sarcopenia Muscle. (2020) 11:1447–58. 10.1002/jcsm.12611 32856431 PMC7749612

[133] Shinmura K Tamaki K Sano M Murata M Yamakawa H Ishida H. Impact of long-term caloric restriction on cardiac senescence: caloric restriction ameliorates cardiac diastolic dysfunction associated with aging. J Mol Cell Cardiol. (2011) 50:117–27. 10.1016/j.yjmcc.2010.10.018 20977912

[134] Weir HJ Yao P Huynh FK Escoubas CC Goncalves RL Burkewitz K. Dietary restriction and Ampk increase lifespan *via* mitochondrial network and peroxisome remodeling. Cell Metab. (2017) 26:884–96.e5. 10.1016/j.cmet.2017.09.024 29107506 PMC5718936

[135] Kobiyama K Ley K. Atherosclerosis. Circ Res. (2018) 123:1118–20. 10.1161/CIRCRESAHA.118.313816 30359201 PMC6298754

[136] Mills EL Kelly B O'Neill LAJ. Mitochondria are the powerhouses of immunity. Nat Immunol. (2017) 18:488–98. 10.1038/ni.3704 28418387

[137] Tyrrell DJ Blin MG Song J Wood SC Zhang M Beard DA. Age-associated mitochondrial dysfunction accelerates atherogenesis. Circ Res. (2020) 126:298–314. 10.1161/CIRCRESAHA.119.315644 31818196 PMC7006722

[138] Grazioli S Pugin J. Mitochondrial damage-associated molecular patterns: from inflammatory signaling to human diseases. Front Immunol. (2018) 9:832. 10.3389/fimmu.2018.00832 29780380 PMC5946030

[139] Yu E Calvert PA Mercer JR Harrison J Baker L Figg NL. Mitochondrial DNA damage can promote atherosclerosis independently of reactive oxygen species through effects on smooth muscle cells and monocytes and correlates with higher-risk plaques in humans. Circulation. (2013) 128:702–12. 10.1161/CIRCULATIONAHA.113.002271 23841983

[140] Khodzhaeva V Schreiber Y Geisslinger G Brandes RP Brüne B Namgaladze D. Mitofusin 2 deficiency causes pro-inflammatory effects in human primary macrophages. Front Immunol. (2021) 12:723683. 10.3389/fimmu.2021.723683 34456930 PMC8397414

[141] Salnikova D Orekhova V Grechko A Starodubova A Bezsonov E Popkova T. Mitochondrial dysfunction in vascular wall cells and its role in atherosclerosis. Int J Mol Sci. (2021) 22:8990. 10.3390/ijms22168990 34445694 PMC8396504

[142] Suárez-Rivero JM Pastor-Maldonado CJ Povea-Cabello S Álvarez-Córdoba M Villalón-García I Talaverón-Rey M. From mitochondria to atherosclerosis: the inflammation path. Biomedicines. (2021) 9:258. 10.3390/biomedicines9030258 33807807 PMC8000234

[143] Wang Q Zhang M Torres G Wu S Ouyang C Xie Z. Metformin suppresses diabetes-accelerated atherosclerosis *via* the inhibition of Drp1-mediated mitochondrial fission. Diabetes. (2017) 66:193–205. 10.2337/db16-0915 27737949 PMC5204316

[144] Bennett MR Sinha S Owens GK. Vascular smooth muscle cells in atherosclerosis. Circ Res. (2016) 118:692–702. 10.1161/CIRCRESAHA.115.306361 26892967 PMC4762053

[145] Peng W Cai G Xia Y Chen J Wu P Wang Z. Mitochondrial dysfunction in atherosclerosis. DNA Cell Biol. (2019) 38:597–606. 10.1089/dna.2018.4552 31095428

[146] Cooper HA Cicalese S Preston KJ Kawai T Okuno K Choi ET. Targeting mitochondrial fission as a potential therapeutic for abdominal aortic aneurysm. Cardiovasc Res. (2021) 117:971–82. 10.1093/cvr/cvaa133 32384150 PMC7898955

[147] Chen KH Guo X Ma D Guo Y Li Q Yang D. Dysregulation of Hsg triggers vascular proliferative disorders. Nat Cell Biol. (2004) 6:872–83. 10.1038/ncb1161 15322553

[148] Guo X Chen KH Guo Y Liao H Tang J Xiao RP. Mitofusin 2 triggers vascular smooth muscle cell apoptosis *via* mitochondrial death pathway. Circ Res. (2007) 101:1113–22. 10.1161/CIRCRESAHA.107.157644 17901359

[149] Poznyak AV Nikiforov NG Wu WK Kirichenko TV Orekhov AN. Autophagy and mitophagy as essential components of atherosclerosis. Cells. (2021) 10:443. 10.3390/cells10020443 33669743 PMC7922388

[150] Razani B Feng C Coleman T Emanuel R Wen H Hwang S. Autophagy links inflammasomes to atherosclerotic progression. Cell Metab. (2012) 15:534–44. 10.1016/j.cmet.2012.02.011 22440612 PMC3322320

[151] Zhang X Sergin I Evans TD Jeong SJ Rodriguez-Velez A Kapoor D. High-protein diets increase cardiovascular risk by activating macrophage Mtor to suppress mitophagy. Nat Metabo. (2020) 2:110–25. 10.1038/s42255-019-0162-4 32128508 PMC7053091

[152] Sergin I Bhattacharya S Emanuel R Esen E Stokes CJ Evans TD. Inclusion bodies enriched for P62 and polyubiquitinated proteins in macrophages protect against atherosclerosis. Sci Signal. (2016) 9:ra2. 10.1126/scisignal.aad5614 26732762 PMC5023144

[153] Grootaert MO da Costa Martins PA Bitsch N Pintelon I De Meyer GR Martinet W. Defective autophagy in vascular smooth muscle cells accelerates senescence and promotes neointima formation and atherogenesis. Autophagy. (2015) 11:2014–32. 10.1080/15548627.2015.1096485 26391655 PMC4824610

[154] Torisu K Singh KK Torisu T Lovren F Liu J Pan Y. Intact endothelial autophagy is required to maintain vascular lipid homeostasis. Aging Cell. (2016) 15:187–91. 10.1111/acel.12423 26780888 PMC4717267

[155] Piché ME Tchernof A Després JP. Obesity phenotypes, diabetes, and cardiovascular diseases. Circ Res. (2020) 126:1477–500. 10.1161/CIRCRESAHA.120.316101 32437302

[156] Ortega FB Lavie CJ Blair SN. Obesity and cardiovascular disease. Circ Res. (2016) 118:1752–70. 10.1161/CIRCRESAHA.115.306883 27230640

[157] Gao CL Zhu C Zhao YP Chen XH Ji CB Zhang CM. Mitochondrial dysfunction is induced by high levels of glucose and free fatty acids in 3t3-L1 adipocytes. Mol Cell Endocrinol. (2010) 320:25–33. 10.1016/j.mce.2010.01.039 20144685

[158] de Mello AH Costa AB Engel JDG Rezin GT. Mitochondrial dysfunction in obesity. Life Sci. (2018) 192:26–32. 10.1016/j.lfs.2017.11.019 29155300

[159] Keller MP Attie AD. Physiological insights gained from gene expression analysis in obesity and diabetes. Annu Rev Nutr. (2010) 30:341–64. 10.1146/annurev.nutr.012809.104747 20415584 PMC5809156

[160] Sparks LM Xie H Koza RA Mynatt R Hulver MW Bray GA. A high-fat diet co-ordinately downregulates genes required for mitochondrial oxidative phosphorylation in skeletal muscle. Diabetes. (2005) 54:1926–33. 10.2337/diabetes.54.7.1926 15983191

[161] Rocha N Bulger DA Frontini A Titheradge H Gribsholt SB Knox R. Human biallelic Mfn2 mutations induce mitochondrial dysfunction, upper body adipose hyperplasia, and suppression of leptin expression. eLife. (2017) 6:e23813. 10.7554/eLife.23813 28414270 PMC5422073

[162] Mancini G Pirruccio K Yang X Blüher M Rodeheffer M Horvath TL. Mitofusin 2 in mature adipocytes controls adiposity and body weight. Cell Rep. (2019) 26:2849–58.e4. 10.1016/j.celrep.2019.02.039 30865877 PMC6876693

[163] Bach D Naon D Pich S Soriano FX Vega N Rieusset J. Expression of Mfn2, the charcot-marie-tooth neuropathy type 2a gene, in human skeletal muscle: effects of type 2 diabetes, obesity, weight loss, and the regulatory role of tumor necrosis factor alpha and interleukin-6. Diabetes. (2005) 54:2685–93. 10.2337/diabetes.54.9.2685 16123358

[164] Pich S Bach D Briones P Liesa M Camps M Testar X. The charcot-marie-tooth type 2a gene product, Mfn2, up-regulates fuel oxidation through expression of oxphos system. Hum Mol Genet. (2005) 14:1405–15. 10.1093/hmg/ddi149 15829499

[165] Tsushima K Bugger H Wende AR Soto J Jenson GA Tor AR. Mitochondrial reactive oxygen species in lipotoxic hearts induce post-translational modifications of Akap121, Drp1, and Opa1 that promote mitochondrial fission. Circ Res. (2018) 122:58–73. 10.1161/CIRCRESAHA.117.311307 29092894 PMC5756120

[166] Finocchietto P Perez H Blanco G Miksztowicz V Marotte C Morales C. Inhibition of mitochondrial fission by Drp-1 blockade by short-term leptin and Mdivi-1 treatment improves white adipose tissue abnormalities in obesity and diabetes. Pharmacol Res. (2021) 178:106028. 10.1016/j.phrs.2021.106028 34896541

[167] Tong M Saito T Zhai P Oka SI Mizushima W Nakamura M. Alternative mitophagy protects the heart against obesity-associated cardiomyopathy. Circ Res. (2021) 129:1105–21. 10.1161/CIRCRESAHA.121.319377 34724805

[168] Ren J Sun M Zhou H Ajoolabady A Zhou Y Tao J. Fundc1 interacts with Fbxl2 to govern mitochondrial integrity and cardiac function through an Ip3r3-dependent manner in obesity. Sci Adv. (2020) 6:eabc8561. 10.1126/sciadv.abc8561 32938669 PMC7494344

[169] Fu T Xu Z Liu L Guo Q Wu H Liang X. Mitophagy directs muscle-adipose crosstalk to alleviate dietary obesity. Cell Rep. (2018) 23:1357–72. 10.1016/j.celrep.2018.03.127 29719250

[170] Cho YK Son Y Saha A Kim D Choi C Kim M. Stk3/Stk4 signalling in adipocytes regulates mitophagy and energy expenditure. Nat Metab. (2021) 3:428–41. 10.1038/s42255-021-00362-2 33758424

[171] Shao D Kolwicz SC Jr Wang P Roe ND Villet O Nishi K. Increasing fatty acid oxidation prevents high-fat diet-induced cardiomyopathy through regulating parkin-mediated mitophagy. Circulation. (2020) 142:983–97. 10.1161/CIRCULATIONAHA.119.043319 32597196 PMC7484440

[172] Frank S Gaume B Bergmann-Leitner ES Leitner WW Robert EG Catez F. The role of dynamin-related protein 1, a mediator of mitochondrial fission, in apoptosis. Dev Cell. (2001) 1:515–25. 10.1016/S1534-5807(01)00055-7 11703942

[173] Deng Y Li S Chen Z Wang W Geng B Cai J. Mdivi-1, a mitochondrial fission inhibitor, reduces angiotensin-II- induced hypertension by mediating VSMC phenotypic switch. Biomed Pharmacother. (2021) 140:111689. 10.1016/j.biopha.2021.111689 34004510

[174] Aishwarya R Alam S Abdullah CS Morshed M Nitu SS Panchatcharam M. Pleiotropic effects of Mdivi-1 in altering mitochondrial dynamics, respiration, and autophagy in cardiomyocytes. Redox Biol. (2020) 36:101660. 10.1016/j.redox.2020.101660 32750667 PMC7396909

[175] Ding J Zhang Z Li S Wang W Du T Fang Q. Mdivi-1 alleviates cardiac fibrosis post myocardial infarction at infarcted border zone, possibly *via* inhibition of Drp1-activated mitochondrial fission and oxidative stress. Arch Biochem Biophys. (2022) 718:109147. 10.1016/j.abb.2022.109147 35143784

[176] Duan C Wang L Zhang J Xiang X Wu Y Zhang Z. Mdivi-1 attenuates oxidative stress and exerts vascular protection in ischemic/hypoxic injury by a mechanism independent of Drp1 Gtpase activity. Redox Biol. (2020) 37:101706. 10.1016/j.redox.2020.101706 32911435 PMC7490562

[177] Lee H Ha TY Jung CH Nirmala FS Park SY Huh YH. Mitochondrial dysfunction in skeletal muscle contributes to the development of acute insulin resistance in mice. J Cachexia Sarcopenia Muscle. (2021) 12:1925–39. 10.1002/jcsm.12794 34605225 PMC8718067

[178] Kugler BA Deng W Duguay AL Garcia JP Anderson MC Nguyen PD. Pharmacological inhibition of dynamin-related protein 1 attenuates skeletal muscle insulin resistance in obesity. Physiol Rep. (2021) 9:e14808. 10.14814/phy2.14808 33904649 PMC8077121

[179] Ma S Dong Z. Melatonin attenuates cardiac reperfusion stress by improving Opa1-related mitochondrial fusion in a Yap-hippo pathway-dependent manner. J Cardiovasc Pharmacol. (2019) 73:27–39. 10.1097/FJC.0000000000000626 30418242 PMC6319588

[180] Chen WR Zhou YJ Yang JQ Liu F Wu XP Sha Y. Melatonin attenuates calcium deposition from vascular smooth muscle cells by activating mitochondrial fusion and mitophagy *via* an Ampk/Opa1 signaling pathway. Oxid Med Cell Longev. (2020) 2020:5298483. 10.21203/rs.2.17699/v2 32377301 PMC7196154

[181] Zhou H Zhang Y Hu S Shi C Zhu P Ma Q. Melatonin protects cardiac microvasculature against ischemia/reperfusion injury *via* suppression of mitochondrial fission-Vdac1-Hk2-Mptp-mitophagy axis. J Pineal Res. (2017) 63:443. 10.1111/jpi.12413 28398674 PMC5518188

[182] Ding M Feng N Tang D Feng J Li Z Jia M. Melatonin prevents Drp1-mediated mitochondrial fission in diabetic hearts through Sirt1-Pgc1α pathway. J Pineal Res. (2018) 65:e12491. 10.1111/jpi.12491 29575122 PMC6099285

[183] Bai Y Yang Y Gao Y Lin D Wang Z Ma J. Melatonin post-conditioning ameliorates anoxia/reoxygenation injury by regulating mitophagy and mitochondrial dynamics in a Sirt3-dependent manner. Eur J Pharmacol. (2021) 904:174157. 10.1016/j.ejphar.2021.174157 33971181

[184] Chang X Zhang T Liu D Meng Q Yan P Luo D. Puerarin attenuates Lps-induced inflammatory responses and oxidative stress injury in human umbilical vein endothelial cells through mitochondrial quality control. Oxid Med Cell Longev. (2021) 2021:6659240. 10.1155/2021/6659240 33728025 PMC7937474

[185] Yang YL Li J Liu K Zhang L Liu Q Liu B. Ginsenoside Rg5 increases cardiomyocyte resistance to ischemic injury through regulation of mitochondrial hexokinase-II and dynamin-related protein 1. Cell Death Dis. (2017) 8:e2625. 10.1038/cddis.2017.43 28230856 PMC5386487

[186] Jiang L Yin X Chen YH Chen Y Jiang W Zheng H. Proteomic analysis reveals ginsenoside Rb1 attenuates myocardial ischemia/reperfusion injury through inhibiting Ros production from mitochondrial complex I. Theranostics. (2021) 11:1703–20. 10.7150/thno.43895 33408776 PMC7778584

[187] Abudureyimu M Yu W Cao RY Zhang Y Liu H Zheng H. Berberine promotes cardiac function by upregulating Pink1/Parkin-mediated mitophagy in heart failure. Front Physiol. (2020) 11:565751. 10.3389/fphys.2020.565751 33101051 PMC7546405

[188] Song N Jia L Cao H Ma Y Chen N Chen S. Gypenoside inhibits endothelial cell apoptosis in atherosclerosis by modulating mitochondria through Pi3k/Akt/Bad pathway. Biomed Res Int. (2020) 2020:2819658. 10.1155/2020/2819658 32685460 PMC7327587

[189] Cui L Li Z Chang X Cong G Hao L. Quercetin attenuates vascular calcification by inhibiting oxidative stress and mitochondrial fission. Vascul Pharmacol. (2017) 88:21–9. 10.1016/j.vph.2016.11.006 27932069

[190] Chen C Huang J Shen J Bai Q. Quercetin improves endothelial insulin sensitivity in obese mice by inhibiting Drp1 phosphorylation at serine 616 and mitochondrial fragmentation. Acta Biochim Biophys Sin. (2019) 51:1250–7. 10.1093/abbs/gmz127 31781748

[191] Li F Li D Tang S Liu J Yan J Chen H. Quercetin protects H9c2 cardiomyocytes against oxygen-glucose deprivation/reoxygenation-induced oxidative stress and mitochondrial apoptosis by regulating the Erk1/2/Drp1 signaling pathway. Evid Based Complement Alternat Med. (2021) 2021:7522175. 10.1155/2021/7522175 34457029 PMC8390138

[192] Chang X Zhang T Meng Q Shiyuan W Yan P Wang X. Quercetin improves cardiomyocyte vulnerability to hypoxia by regulating Sirt1/Tmbim6-related mitophagy and endoplasmic reticulum stress. Oxid Med Cell Longev. (2021) 2021:5529913. 10.1155/2021/5529913 33859776 PMC8024107

[193] Chang X Zhang T Wang J Liu Y Yan P Meng Q. Sirt5-related desuccinylation modification contributes to quercetin-induced protection against heart failure and high-glucose-prompted cardiomyocytes injured through regulation of mitochondrial quality surveillance. Oxid Med Cell Longev. (2021) 2021:5876841. 10.1155/2021/5876841 34603599 PMC8486530

[194] Chang X Yao S Wu Q Wang Y Liu J Liu R. Tongyang huoxue decoction (Tyhx) ameliorating hypoxia/reoxygenation-induced disequilibrium of calcium homeostasis and redox imbalance *via* regulating mitochondrial quality control in sinoatrial node cells. Oxid Med Cell Longev. (2021) 2021:3154501. 10.1155/2021/3154501 34422207 PMC8373484

[195] Liu R Chang X Li J Shunyu Y. Zishen huoxue recipe protecting mitochondrial function of hypoxic/reoxygenated myocardial cells through Mtorc1 signaling pathway. Evid Based Complement Alternat Med. (2020) 2020:8327307. 10.1155/2020/8327307 32802135 PMC7403935

